# Chinese herbal formulas as adjuncts to antihistamines in chronic spontaneous urticaria: a network meta-analysis of efficacy, recurrence, and safety

**DOI:** 10.3389/fphar.2026.1718329

**Published:** 2026-04-22

**Authors:** Haiying Lv, Haoting Zhu, Huixia Mo, Jie Guo, Chuanjian Lu, Jingwen Deng

**Affiliations:** 1 Second Clinical Medical College, Guangzhou University of Chinese Medicine, Guangzhou, China; 2 State Key Laboratory of Dampness Syndrome of Chinese Medicine, The Second Affiliated Hospital of Guangzhou University of Chinese Medicine, Guangzhou, China; 3 Guangdong-Hong Kong-Macau Joint Lab on Chinese Medicine and Immune Disease Research, Guangzhou University of Chinese Medicine, Guangzhou, China; 4 State Key Laboratory of Traditional Chinese Medicine Syndrome, The Second Affiliated Hospital of Guangzhou University of Chinese Medicine, Guangzhou, China

**Keywords:** chronic spontaneous urticaria, classical TCM formulas, meta-analysis, second-generation H1-antagonists, traditional Chinese medicine

## Abstract

**Background:**

Chronic spontaneous urticaria (CSU) presents treatment challenges despite second-generation antihistamines (sgAHs) serving as the first-line therapy.

**Purpose:**

The aim of this network meta-analysis is to systematically evaluate the efficacy hierarchy and safety profile of classical traditional Chinese medicine (TCM) formulas used as adjuncts to antihistamines.

**Methods:**

We systematically evaluated 50 randomized controlled trials involving 5,814 patients using both Bayesian and frequentist network meta-analyses (NMAs) to compare the efficacy, recurrence, and safety of sgAHs alone or in combination with classical TCM formulas. The primary outcome was clinical improvement based on the Symptom Score Reducing Index (SSRI), with secondary outcomes including recurrence rates and adverse events (AEs). Risk of bias was assessed using RoB 2.0, and evidence certainty was evaluated using the CINeMA framework. Bayesian and frequentist NMA approaches were employed. Sensitivity analyses were conducted based on the study quality and prescription modifications.

**Results:**

Most combination regimens of classical TCM formulas were superior to antihistamine monotherapy for both SSRI ≥90% and ≥60% outcomes. Combinations involving *Danggui Yinzi, Yupingfeng powder,* and *Guizhi decoction* consistently ranked among the top in terms of efficacy. Among these regimens, loratadine or ebastine combined with *Danggui Yinzi* demonstrated the most pronounced reduction in recurrence risk, with the effect being more evident in the long-course subgroup (>12 weeks). Certain combination regimens, such as cetirizine combined with *Danggui Yinzi* and loratadine combined with *Guizhi decoction*, were associated with a reduced risk of AEs. However, no evaluated treatment showed a statistically significant advantage over cetirizine monotherapy in reducing IgE or IL-4 levels. The certainty of evidence for efficacy outcomes was predominantly moderate, whereas the overall certainty for safety outcomes was relatively low.

**Conclusion:**

In CSU management, classical TCM formulas, particularly *Danggui Yinzi, Yupingfeng powder,* and *Guizhi decoction,* may improve remission and reduce recurrence when combined with sgAHs without increasing the incidence of AEs. Further high-quality head-to-head trials are needed to confirm these findings.

**Systematic Review Registration:**

https://www.crd.york.ac.uk/PROSPERO, identifier CRD420251022634.

## 1 Introduction

Chronic spontaneous urticaria (CSU) is a chronic inflammatory skin disorder characterized by the presence of wheals, angioedema, or both for at least 6 weeks (Zuberbier et al., 2018). Some patients may develop mucosal symptoms such as abdominal pain or laryngeal edema. As a common disease affecting individuals of all ages (Gonçalo et al., 2021), CSU has a slightly higher incidence rate among women than among men (Fricke et al., 2020). Epidemiological data indicate that its prevalence has increased by 2–10 times over the past decade. The highest prevalence rate in Asian populations is 1.4%, which is significantly higher than that in Europe and North America (Fricke et al., 2020). The global lifetime prevalence rate of chronic urticaria (CU) is estimated at 4.4% (Gonçalo et al., 2021). This disease significantly reduces the quality of life by disrupting sleep, daily activities, occupational performance, and physical exercise (Maurer et al., 2017), while also imposing a heavy financial burden, with the annual medical expenditures in Europe and the United States reaching approximately $15,550 (Gammeri et al., 2023). CSU has a multifactorial etiology involving genetic predisposition, autoimmune dysregulation, infections, and psychological stress (Centre for Urticaria Research et al., 2022; Kaplan et al., 2023), with no currently available curative treatment. According to the EAACI/GALEN/EDF/AAACI International Guidelines (Zuberbier et al., 2018), second-generation H1-antihistamines (sgAHs) are recommended as the first-line therapy. However, approximately 50% of patients experience suboptimal responses, requiring quadruple-dose sgAHs or combination therapy with biologic agents and immunosuppressants (Zuberbier et al., 2018). Studies indicate that while drug treatments can alleviate pruritus in some patients, achieving complete symptom control remains challenging (Guillén-Aguinaga et al., 2016), and recurrence often occurs upon discontinuation of therapy. This therapeutic landscape underscores the urgent need for alternative treatments.

Traditional Chinese medicine (TCM) provides unique therapeutic advantages for CSU. TCM classifies urticaria into Yin Zhen (hidden rash) and Feng Zhen (wind rash) types. The clinical manifestations vary depending on the severity of the disease and the pathogenesis, and treatment must follow the principle of syndrome differentiation and treatment. Classical TCM formulas, as a core treatment method, are derived from ancient medical records. Their formulations are rigorously structured and have undergone centuries of clinical validation, and they are the benchmark for clinical efficacy and precise treatment in TCM to this day. Recent studies have shown that combining classic prescriptions with antihistamines can significantly improve the efficacy, reduce recurrence rates, and improve patients’ quality of life (Yang et al., 2018). Some studies have also indicated that the efficacy of classic TCM formulas in the treatment of CU, although worthy of attention, is poorly documented (Chen et al., 2012).

While the evidence for integrating classical TCM formulas with biomedicine shows promising prospects, significant limitations remain. There is a severe lack of randomized controlled trials (RCTs) that directly compare the efficacy of different classical prescriptions, and existing studies are mostly small-scale, low-quality trials with common problems such as irregular randomization, lack of blinding implementation, and insufficient long-term follow-up data. This study aims to employ a network meta-analysis (NMA) approach to integrate RCT data on classical TCM formulas combined with sgAHs, achieving two objectives: (1) To establish a ranking system of treatment efficacy for combined regimens in terms of response rates, recurrence rates, and other outcomes and (2) to compare the safety profiles of different combination therapies. This study provides evidence supporting the effective integration of TCM with sgAHs for treating CSU.

## 2 Methods

This systematic review and NMA was designed in strict adherence to the Preferred Reporting Items for Systematic Reviews and Meta-Analyses (PRISMA) guidelines (Hutton et al., 2015). The study protocol was prospectively registered with the International Systematic Review registry PROSPERO (registration number: CRD420251022634).

### 2.1 Search strategy

The entire process of this study strictly followed PRISMA guidelines. An open search strategy combined with structured screening criteria was used to systematically evaluate the efficacy and safety of clinical interventions for CSU, focusing on the evidence integration of the therapeutic effects of classical TCM formulas. Computer searches covered the following databases: China National Knowledge Infrastructure (CNKI), WanFang Data, VIP database, Chinese Clinical Trial Registry (ChiCTR), Medline, Embase, and Cochrane Library, Clinical Trial. The search timeframe spanned from database inception to October 2024. This study employed search terms such as “urticaria” and “clinical trials.” The retrieval strategy integrated controlled vocabulary with free-text combinations, specifically utilizing a nonrestrictive intervention approach—avoiding predefined terms like “classic formulas” or specific prescription names—to circumvent potential literature omissions arising from historical naming variations, dosage form changes, or spelling variants in TCM formulations. The detailed search strategies are shown in Supplementary Material S1.

### 2.2 Study selection

**FIGURE 1:**
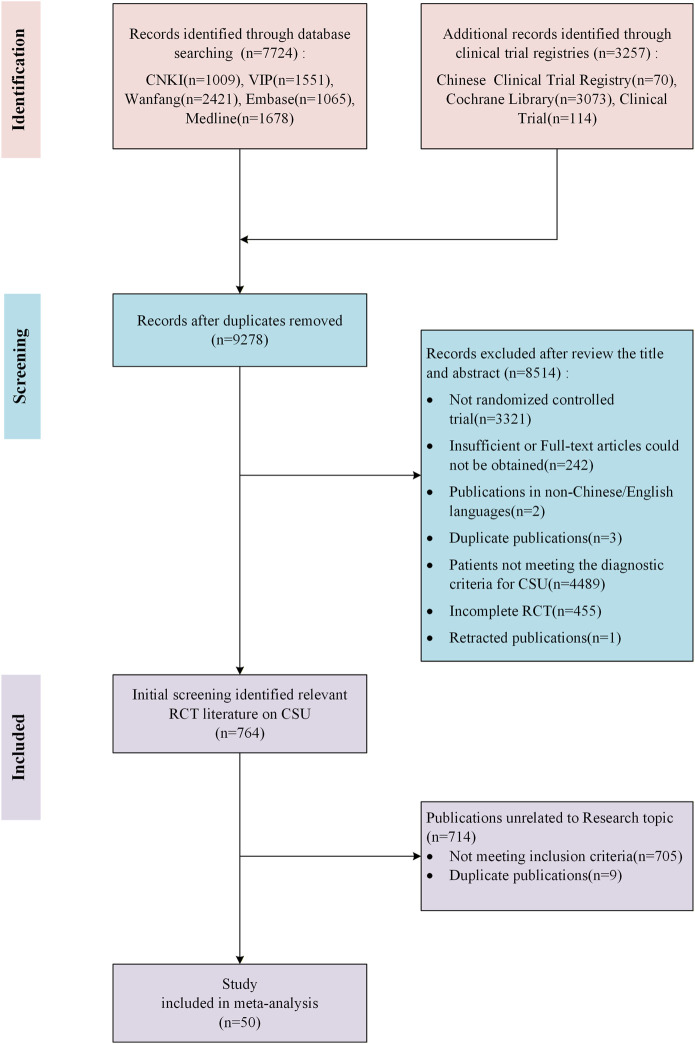
Flow chart of literature screening.

During the initial screening process, we excluded non-journal publications such as book chapters and conference proceedings, focusing only on peer-reviewed research articles. After removing duplicates, we completed the first round of screening by reviewing the titles and abstracts to exclude obviously irrelevant studies. Subsequently, the remaining articles were retrieved in full text and subjected to secondary screening based on predefined criteria. The screening process was independently performed and cross-checked by three researchers (LHY, MHX, and ZHT). Any disagreements were arbitrated by another researcher (DJW). The detailed process is shown in Figure 1.

The inclusion criteria were set as follows: (1) study design: RCT; (2) patient population: diagnosed CSU; (3) intervention measures: the experimental group was various TCM herbal prescriptions with different compositions or dosage forms, and the controls included sgAHs; (4) outcomes: the primary outcome indicator was the effective rate, and the secondary outcome indicators included the recurrence rate and the incidence of adverse reactions.

Our study permitted minor adjustments to the prescriptions during the data synthesis phase. Specifically, we allowed the following: (1) dosage form conversions (e.g., decoction to granule) without composition changes; (2) adjustments of individual herb dosages within ±40% of the original prescription for formulas containing fewer than five herbs and within ±30% for formulas containing five or more herbs based on clinical practice variations, and (3) substitutions of herbs within the same pharmacological category. We conducted a sensitivity analysis excluding studies with any prescription modifications to test the robustness of our results.

Exclusion criteria included the following: (1) non-RCTs, (2) retention of only the most recent publication when multiple studies reported identical patient cohorts, (3) studies lacking sufficient extractable data, (4) investigations involving urticaria complicated with concurrent systemic disorders, and (5) therapeutic interventions deviating from the predefined protocol specifications.

### 2.3 Data extraction

The data extraction protocol included the following parameters: first author, publication year, sample size, TCM syndrome differentiation, study duration, therapeutic interventions, outcome measures, adverse events (AEs), follow-up duration, and recurrence rates.

### 2.4 Assessment of TCM formula standardization

All classical TCM formulas were assessed using the reporting guidelines of the Consensus statement on the Phytochemical Characterization of Medicinal Plant extracts (ConPhyMP) (Heinrich et al., 2022).

### 2.5 Taxonomic validation

All plant species were taxonomically validated through the Kew Garden MPNS portal (http://mpns.kew.org/mpns-portal/).

### 2.6 Quality assessment

Quality assessments of the included studies were conducted independently by two assessors (LHY and ZHT). Disagreements were resolved through discussion or by consultation with a third researcher (DJW). The evaluation tool, based on the Cochrane risk-of-bias assessment tool version 2.0 (RoB 2.0) (Sterne et al., 2019), assessed the quality of RCTs across five dimensions: randomization process, deviations from intended interventions, missing outcome data, measurement of the outcome, and selection of the reported result.

Since the RoB 2.0 tool primarily focuses on the internal validity of RCTs, we supplemented its assessment criteria to account for the specific characteristics of the included studies. In addition to evaluating the clinical trial design, we introduced external validity indicators, including the following: whether the study was conducted by a single author, whether funding support was provided, whether a validated disease severity scoring system was used, whether an ethics approval number was documented, whether a clinical trial registration number was provided, and whether the TCM syndrome patterns were reported. To synthesize these assessments, we assigned each RCT a composite quality score by combining the RoB 2.0 evaluation with the external validity indicators. This approach provided a holistic appraisal of both the internal and external sources of bias.

### 2.7 Credibility assessment

To assess the credibility of evidence for each comparison in our NMA, we applied the Confidence in Network Meta-Analysis (CINeMA) framework (Nikolakopoulou et al., 2020). CINeMA is a web-based tool developed by the Cochrane Comparing Multiple Interventions Methods Group as an adaptation of the Grading of Recommendations, Assessment, Development, and Evaluation (GRADE) approach specifically for network meta-analyses (Papakonstantinou et al., 2020). Following the recommended guidelines, we evaluated the evidence quality across six key domains for each comparison: within-study risk of bias, reporting bias, indirectness, imprecision, heterogeneity, and incoherence (also referred to as inconsistency). This systematic approach allowed us to provide transparent and standardized assessments of the certainty in our NMA findings.

### 2.8 Network meta-analysis

We conducted a NMA using both Bayesian and frequentist approaches in R (version 4.4.1) with the BUGSnet and netmeta packages. The Bayesian NMA employed Markov chain Monte Carlo (MCMC) simulations with vague priors, running three chains with a burn-in of 1,000 iterations, followed by 10,000 sampling iterations and 1,000 adaptations. The network geometry was visualized using interactive network plots, while the model fit was assessed through leverage plots, total residual deviance, and the deviance information criterion (DIC). The choice between the fixed-effects and random-effects models was determined by selecting the model with the lower DIC value, indicating better fit to the data (Supplementary Figure S1). To assess the robustness, we compared Bayesian results with frequentist estimates. For Bayesian analyses, the median effect estimates were reported with 95% credible intervals (CrIs), while the frequentist results used point estimates with 95% confidence intervals (CIs). To combine laboratory measures reported as mean and standard deviation (SD) across studies with different units, we computed the standardized mean difference (SMD) and its standard error (SE) for each study. The SMD was estimated using Hedges’ g to adjust for small-sample bias. Statistically significant comparisons (intervals excluding the null value) were highlighted to facilitate clinical interpretation. The statistical significance was set at P < 0.05.

### 2.9 Effect measures

Treatment rankings were assessed using the surface under the cumulative ranking curve (SUCRA), representing the probability of a given treatment being the optimal choice. The primary efficacy outcome was the clinical improvements, which was defined based on the Symptom Score Reducing Index (SSRI) (Sharma et al., 2014; Chen et al., 2024). The SSRI was calculated using the following formula:

SSRI (%) = [(baseline symptom score − post-treatment symptom score)/baseline symptom score] × 100%

Treatment response was categorized according to the SSRI value, where SSRI ≥90% indicated clinical cure/significant improvement and SSRI ≥60% indicated effective treatment response. The improvement rate was calculated as the proportion of patients achieving an SSRI ≥60% (responders) relative to the total cohort size, and it was expressed as a percentage.

The secondary outcome was the recurrence rate. Safety outcomes were evaluated based on the incidence of AEs. To complement the SUCRA-based rankings, we generated a league table to present all pairwise treatment comparisons for both the efficacy and safety outcomes. This table displays the effect measures [e.g., risk ratios (RRs) for improvement and odds ratios (ORs) for recurrence events or AEs) with 95% CIs/CrIs, thus allowing direct comparison of the interventions. The league table structure aligns with network geometry, ensuring consistency between visual and numerical results.

### 2.10 Assessment of heterogeneity and inconsistency

Heterogeneity and inconsistency were assessed with both global and local methods. Within-designs Q statistics evaluated homogeneity among the studies with identical comparisons, both globally (across the entire network) and locally (for each pairwise comparison). Between-study variance was quantified using tau-squared (τ^2^), with values < 0.01, 0.01–0.04, and >0.04 indicating low, moderate, and substantial heterogeneity, respectively.

The design-by-treatment interaction model assessed global consistency by decomposing total heterogeneity into within-designs (heterogeneity) and between-designs (inconsistency) components. A nonsignificant between-designs Q test (p > 0.05) indicates consistency between the direct and indirect evidence. Bayesian consistency and inconsistency models were compared using the deviance information criterion (DIC), with ΔDIC <5 indicating no meaningful inconsistency. Leverage plots identified potentially influential data points.

Comparison-adjusted funnel plots assessed small-study effects, along with visual symmetry evaluation and Egger’s regression test (significance at p < 0.10). Contour-enhanced plots distinguished asymmetry from heterogeneity by overlaying statistical significance regions.

### 2.11 Sensitivity analysis

We conducted the following sensitivity analyses to test the robustness of our results: (1) quality-based sensitivity analysis, excluding studies with more than two biases with high risk according to RoB 2.0; (2) composition-based sensitivity analysis, excluding studies with prescription modifications exceeding our predefined “minor adjustments” criteria. Assessment of inconsistency, which refers to the agreement between indirect and direct evidence within a network, was carried out using the inconsistency model.

## 3 Results

### 3.1 Study characteristics

In this NMA, we screened 10,981 records and included 50 RCTs, encompassing a total of 5,814 patients (Figure 1). Among the included studies, 49 were two-arm trials and one was a multiarm study. While the network allowed for 230 possible pairwise comparisons, direct evidence was available for only 22 comparisons, highlighting the value of NMA in synthesizing evidence across the treatment network. The interventions primarily consisted of combinations of three antihistamines (cetirizine, ebastine, and loratadine) with various TCM formulas, enabling a comprehensive assessment of both sgAH monotherapy and integrated treatment approaches. The characteristics of each trial are summarized in Table 1. A detailed ConPhyMP assessment report of these 50 studies is provided in Supplementary Appendix Table S1 and Supplementary Material S2. The report of taxonomic validation is available in Supplementary Appendix Table S2.

**TABLE 1:** Characteristics of the included studies.

Study ID	TCM-SP	Number of case	Mean age (years)	Male proportion (%)	Intervention measures		Treatment course (weeks)	Outcome measures
		T vs. C	T vs. C	T vs. C	T	C		
Li and Ma (2021)	—	30 vs. 30	(28.90 ± 2.78) vs. (28.80 ± 2.27)	​	Ebastine + *Yupingfeng powder*	Ebastine	10	1, 2, 4
Fu and Liu (2020)	—	40 vs. 40	(34.17 ± 4.15) vs. (33.88 ± 4.23)	57.5% vs. 50.0%	Cetirizine + *Yupingfeng powder*	Cetirizine	4	7, 9
Wang et al. (2019)	—	50 vs. 50	(39.12 ± 5.37) vs. (39.21 ± 5.30)	58.0% vs. 60.0%	Loratadine + *Yupingfeng powder*	Loratadine	4	2, 4
Wang and Zhang (2019)	—	35 vs. 35	(31.65 ± 9.26) vs. (31.69 ± 9.25)	57.1% vs. 60.0%	Loratadine + *Yupingfeng powder*	Loratadine	1	7, 8, 9
Li et al. (2018)	*Exterior Deficiency with Insecurity Syndrome*	40 vs. 40	(35.2 ± 5.3) vs. (35.5 ± 5.4)	37.5% vs. 47.5%	Cetirizine + *Yupingfeng powder*	Cetirizine	4	1, 6, 7, 8
Wang and Zhang (2018)	—	60 vs. 60	(34.3 ± 10.1) vs. (35.8 ± 10.4)	65.0% vs. 60.0%	Cetirizine + *Yupingfeng powder*	Cetirizine	4	1, 4, 5, 8, 9
Cai (2016)	—	60 vs. 60	34.6 vs. 35.4	46.6% vs. 46.6%	Ebastine + *Yupingfeng powder*s	Ebastine	4	7, 8
Xu (2016)	—	40 vs. 40	(36.9 ± 5.7) vs. (35.2 ± 5.1)	37.5% vs. 35.0%	Cetirizine + *Yupingfeng powder*	Cetirizine	4	7, 8, 9
Zhao (2015)	—	134 vs. 134	(56.4 ± 31.5)	52.20%	Ebastine + *Yupingfeng powder*	Ebastine	4	6, 7, 8
Fu et al. (2015)	—	100 vs. 110	(35.0 ± 3.0) vs. (36.5 ± 2.5)	58.0% vs. 54.5%	Loratadine + *Yupingfeng powder*	Loratadine	8	2, 4, 7
Tian and Huang (2015)	—	86 vs. 86	36.5 vs. missing data	48.8% vs. 58.1%	Loratadine + *Yupingfeng powder*s	Loratadine	4	4, 6, 7, 8
Niu (2015)	—	64 vs. 64	35.2 vs. 35.7	51.6% vs. 50.0%	Cetirizine + *Yupingfeng powder*	Cetirizine	8	6, 7, 8, 9
Jiang (2014)	—	42 vs. 40	(34.5 ± 10.6) vs. (37.2 ± 12.9)	52.3% vs. 50.0%	Ebastine + *Yupingfeng powder*	Ebastine	4	8
Chen and He (2013)	—	24 vs. 18	(41.7 ± 17.2) vs. (41.2 ± 16.9)	45.8% vs. 50.0%	Ebastine + *Yupingfeng powder*	Ebastine	4	6, 7, 8
Xie (2011)	—	62 vs. 60	(25.5 ± 18.2) vs. (26.8 ± 19.1)	54.8% vs.53.3%	Cetirizine + *Yupingfeng powder*	Cetirizine	4	6, 8
Zhan et al. (2024)	*Blood Deficiency with Wind* — *Dryness Syndrome*	45 vs. 45	(42.53 ± 2.58) vs. (42.52 ± 2.60).	51.11% vs. 53.33%	Loratadine + *Danggui Yinzi*	Loratadine	8	4, 7
Cao (2019)	*Blood Deficiency with Wind*—*Dryness Syndrome*	75 vs. 75	(46.7 ± 4.4)vs. (47.2 ± 4.6)	56.0% vs. 61.3%	Cetirizine + *Danggui Yinzi*	Cetirizine	4	1
Huang and Duan (2018)	—	53 vs. 53	(31.14 ± 4.59) vs. (30.72 ± 4.78)	50.9% vs. 52.8%	Ebastine + *Danggui Yinzi*	Ebastine	4	4, 6, 7, 8, 9
Hu et al. (2016)	*Blood Deficiency with Wind* - *Dryness Syndrome*	74 vs. 70	(46.13 ± 19.40) vs. (45.34 ± 18.67)	43.2% vs. 48.5%	Cetirizine + *Danggui Yinzi*	Cetirizine	4	4, 5
Liu (2016)	—	49 vs. 49	(34.5 ± 6.7)	47.96%	Cetirizine + *Danggui Yinzi*	Cetirizine	4	2, 7
Cheng (2016)	*Blood Deficiency with Wind-Dryness Syndrome*	50 vs. 50	(37.09 ± 4.82) vs. (36.23 ± 4.65)	60% vs. 58%	Loratadine + *Danggui Yinzi*	Loratadine	8	1, 4
Shi et al. (2013)	—	25 vs. 25	(35.37 ± 13.54) vs. (36.63 ± 13.98)	44% vs. 48%	Cetirizine + *Danggui Yinzi*	Cetirizine	4	1, 2, 3, 7, 8, 9
Zhang and Lin (2011)	*Blood Deficiency with Wind-Dryness Syndrome*	44 vs. 44	26.8 vs. 27.1	47.73% vs. 50%	Cetirizine + *Danggui Yinzi*	Cetirizine	4	3, 6, 7
Xu (2024)	—	49 vs. 49	(35.78 ± 9.87) vs. (36.24 ± 10.15)	20.41% vs. 36.73%	Cetirizine + *Guizhi decoction*	Cetirizine	2	2, 4, 8, 9
Li (2021)	*Wind-Cold Constraining Exterior Syndrome*	49 vs. 49	(37.01 ± 5.72) vs. (37.58 ± 5.40)	42.86% vs. 38.78%	Ebastine + *Guizhi decoction*	Ebastine	4	2
Zhou (2020)	—	48 vs. 48	(40.03 ± 3.12) vs. (39.94 ± 3.83)	43.75% vs. 45.83%	Loratadine + *Guizhi decoction*	Loratadine	4	2, 8, 9
Wu (2019)	—	45 vs. 44	(39.6 ± 4.2) vs. (39.0 ± 4.5)	53.3% vs. 54.5%	Loratadine + *Guizhi decoction*	Loratadine	4	1
Yu and Shang (2016)	—	30 vs. 30	16 to 62	40.0% vs. 46.7%	Loratadine + *Guizhi decoction*	Loratadine	4	8
Cao (2015)	—	40 vs. 36	(32.3 ± 3.6) vs. (33.3 ± 4.2)	55.0% vs. 57.8%	Loratadine + *Guizhi decoction*	Loratadine	4	5, 6, 8
Yang et al. (2018)	—	37 vs. 37	(41.5 ± 14.09) vs. (39.5 ± 13.92)	75.0% vs. 57.1%	4 weeks prior to treatment: Loratadine + CHM (*Xiaofeng powde*r and *Qingshang Fangfeng decoction*), 4 weeks post-treatment: Loratadine	4 weeks prior to treatment: Cetirizine + CHM placebo (*Xiaofeng powde*r and *Qingshang Fangfeng decoction*), 4 weeks post-treatment: Cetirizine	8	1, 4, 5, 6, 8
Lu (2011)	*Wind Heat Syndrome*	40 vs. 35	—	42.5% vs. 42.8%	Loratadine + *Xiaofeng powde*r	Loratadine	4	—
Bai (2011)	—	34 vs. 34	31.4	47%	Loratadine + *Xiaofeng powde*r	Loratadine	3	1, 7
Wu and Lan (2023)	*Blood Heat Generating Wind Syndrome*	40 vs. 40	(8.94 ± 1.58) vs. (8.96 ± 1.23)	35.90% vs. 39.47%	Cetirizine + *Xiaofeng powde*r	Cetirizine	4	1, 4
Zhang (2023)	—	41 vs. 41	(42.63 ± 2.94) vs. (42.61 ± 2.97)	41.46% vs. 39.02%	Ebastine + *Maxing Shigan decoction*	Ebastine	4	2, 4, 5
Wang et al. (2023)	—	34 vs. 34	(44.02 ± 4.81) vs. (43.19 ± 4.79)	52.9% vs. 55.88%	*Ebastine* + *Danggui Sini decoction*	Ebastine	4	2, 4, 6, 7, 8, 9
Ying (2022)	—	63 vs. 63	(37.52 ± 4.89) vs. (36.53 ± 4.35)	52.38% vs. 55.56%	Ebastine + *Fangfeng Tongsheng decoction*	Ebastine	8	1, 7, 8, 9
Yang et al. (2024)	*Blood Deficiency with Wind-Dryness Syndrome*	38 vs. 38	(36.30 ± 4.03) vs. (36.11 ± 4.01)	52.63% vs. 57.89%	Ebastine + *Fangfeng Xionggui decoction*	Ebastine	8	2, 4, 5, 8, 9
Yang et al. (2021)	*Blood Heat Syndrome*	76 vs. 76	(38.33 ± 5.29) vs. (38.49 ± 5.34)	51.32% vs. 55.26%	Ebastine + *Huanglian Jiedu decoction*	Ebastine	4	2, 4, 8, 9
Mei et al. (2024)	—	31 vs. 31	(38.17 ± 4.36) vs. (39.23 ± 4.49)	51.6% vs. 54.8%	Cetirizine + *Mahuang Lianqiao Chixiaodou decoction*	Cetirizine	4	1, 4, 8, 9
Xu et al. (2019)	*Spleen Deficiency with Dampness Retention Syndrome*	44 vs. 44	(42.64 ± 9.12) vs. (42.87 ± 9.74)	47.73% vs. 40.91%	Ebastine + *Mahuang Fuzi Xixin decoction*	Ebastine	4	2, 4, 5, 7, 8, 9
Mao (2023)	*Spleen Deficiency Syndrome*	55 vs. 55	(43.60 ± 7.60) vs. (44.50 ± 8.20)	58.2% vs. 54.5%	Cetirizine + *Chushi Weiling decoction*	Cetirizine	4	1, 5, 7
Liu X (2020)	—	50 vs. 50	(42.67 ± 3.81) vs. (42.50 ± 3.50)	46% vs. 48%	Ebastine	Cetirizine	4	1, 8, 9
Li et al. (2006)	—	33 vs. 45	(32.5 ± 7.8) vs. (41.9 ± 6.9)	51.5% vs. 57.8%	Loratadine	Cetirizine	4	2, 6, 7, 8, 9
Ou et al. (2005)	—	62 vs. 58	(30.2 ± 8.56) vs. (31.5 ± 8.97)	45.2% vs. 48.3%	Cetirizine	Loratadine	4	8
Zhuang (2005)	—	57 vs. 56	39.33	41.60%	Cetirizine	Loratadine	4	8, 9
Yin et al. (2003)	—	32 vs. 34	36.5 vs. 38 vs.	62.5% vs. 58.8%	Loratadine	Cetirizine	4	2, 6, 7, 8, 9
Lin et al. (2018)	—	60 vs. 60	(36.37 ± 12.76) vs. (36.78 ± 12.21)	33.3% vs. 43.3%	Loratadine	Cetirizine	4	3, 4, 8, 9
Xie et al. (2010)	—	50 vs. 50	(34.5 ± 7.3)	47.50%	Loratadine	Cetirizine	4	8
Xie et al. (2010)	—	50 vs. 50	(34.5 ± 7.3)	47.50%	Ebastine	Cetirizine	4	8
Potter et al. (2009)	—	438 vs. 448	(43.36 ± 15.31) vs. (42.85 ± 14.86)	35.2% vs. 31.7%	Cetirizine	Loratadine	4	1, 2, 8, 9
Lin and Li (2009)	—	76 vs. 47	—	—	Cetirizine	Loratadine	4	8

T, treatment group; C, control group; TCM-SP, traditional Chinese medicine syndrome; 1, UAS score; 2, urticaria symptom scores; 3, symptom score reduce index; 4, blood biomarkers; 5, DLQI score; 6, follow-up duration; 7, recurrence rate; 8, adverse reactions; 9, incidence of adverse reactions.

Among the 13 studies (31.7%) reporting TCM syndrome types, the distribution was as follows: six studies reported *Blood Deficiency with Wind-Dryness Syndrome* (Zhang and Lin, 2011; Cheng, 2016; Hu et al., 2016; Cao, 2019; Yang et al., 2024; Zhan et al., 2024), one study described *Blood Heat Syndrome* (Yang et al., 2021), one study reported *Blood Heat Generating Wind Syndrome *(Wu and Lan, 2023), one study described *Spleen Deficiency Syndrome* (Mao, 2023), one study reported *Spleen Deficiency with Dampness Retention Syndrome* (Xu et al., 2019), one study reported *Wind Heat Syndrome* (Lu, 2011), one study focused on *Wind-Cold Constraining Exterior Syndrome* (Li, 2021), and one study reported *Exterior Deficiency with Insecurity Syndrome* (Yang et al., 2024). In these studies, the following herbal formulas were prescribed for different TCM syndrome types: the *Blood Deficiency with Wind-Dryness Syndrome* was addressed using *Danggui Yinzi* (Zhang and Lin, 2011; Cheng, 2016; Hu et al., 2016; Cao, 2019; Zhan et al., 2024) and *Fangfeng Xionggui decoction* (Yang et al., 2024), while *Exterior Deficiency with Insecurity Syndrome* was treated with *Yupingfeng decoction* (Li et al., 2018). *Wind-Cold Constraining Exterior Syndrome* was treated with *Guizhi decoction* (Li, 2021). *Wind Heat Syndrome* and *Blood Heat Generating Wind Syndrome* both were treated with *Xiaofeng powder* (Lu, 2011; Wu and Lan, 2023). *Blood Heat Syndrome* was managed using *Huanglian Jiedu decoction* (Yang et al., 2021). *Spleen Deficiency with Dampness Retention Syndrome* was managed with *Mahuang Fuzi Xixin decoction* (Xu et al., 2019), and *Spleen Deficiency Syndrome* was treated using *Chushi Weiling decoction* (Mao, 2023). Additionally, 13 studies (Bai, 2011; Cao, 2015; Liu, 2016; Yu and Shang, 2016; Wang and Zhang, 2019; Wu, 2019; Xu et al., 2019; Zhou, 2020; Li, 2021; Li and Ma, 2021; Mao, 2023; Mei et al., 2024; Xu, 2024) (31.7%) documented symptom-driven modifications of herbal decoctions, though there were three overlapping cases among these reports (Xu et al., 2019; Li, 2021; Mao, 2023). These syndrome–formula correspondences represent clinical refinements derived from the physicians' cumulative experience and empirical optimization through clinical practice. The compositions of the formulas included in this study are detailed in Supplementary Appendix Table S2.

Among the 41 studies with integrated treatments, AEs were documented in 15 studies involving *Yupingfeng powder* (four studies, 26.7%). These studies involved the combinations of loratadine combined with *Yupingfeng powder* (Wang and Zhang, 2019) and cetirizine combined with *Yupingfeng powder* (Niu, 2015; Xu, 2016; Wang and Zhang, 2018). Of the eight studies involving *Danggui Yinzi*, AEs were reported in two studies (25.0%), which occurred in combinations with ebastine (Huang and Duan, 2018) and cetirizine (Shi et al., 2013). Among the six studies involving *Guizhi decoction*, two studies (33.3%) reported the incidence of AEs. These studies involved the combinations of cetirizine combined with *Guizhi decoction* (Xu, 2024) and loratadine combined with *Guizhi decoction* (Zhou, 2020). Incidences of AEs were documented for ebastine combined with *Danggui Sini decoction* (Wang et al., 2023), ebastine combined with *Fangfeng Tongsheng decoction* (Ying, 2022), ebastine combined with *Fangfeng Xionggui decoction* (Yang et al., 2024), ebastine combined with *Huanglian Jiedu decoction* (Yang et al., 2021), cetirizine combined with *Mahuang Lianqiao Chixiaodou decoction* (Mei et al., 2024), and cetirizine combined with *Mahuang Fuzi Xixin decoction* (Xu et al., 2019) (each with one study). Although cetirizine combined with *Xiaofeng powder* recorded adverse reactions, the specific incidence data were not provided.

### 3.2 Quality assessment

**FIGURE 2:**
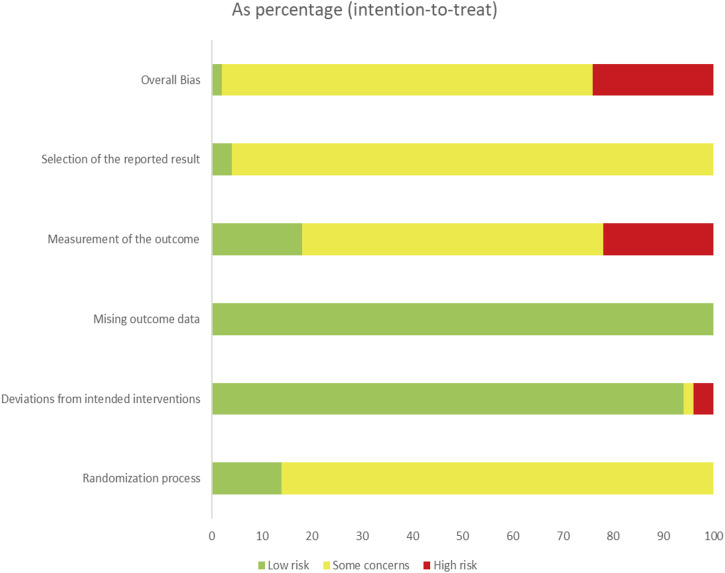
Assessment of risk with the RoB 2.0 tool of the included studies.

We systematically evaluated all included RCTs, comprising 41 studies analyzing classical TCM formulas. The RoB 2.0 tool was employed to assess the methodological quality across five domains, following its standardized evaluation framework for RCTs. (1) Randomization process: 31 studies clearly described randomization, 16 only stated “randomized” without details, and three had unclear procedures. The Baseline characteristics were comparable across groups. (2) Deviations from the intended interventions: no crossover between groups occurred in any of the included studies, and in most trials, the interventions were implemented in accordance with the study protocol, thereby effectively minimizing bias arising from deviations from the intended interventions. (3) Missing outcome data: documentation of participant attrition was identified in only three studies (7.3%), while over 95% outcome data completeness was achieved across all studies. (4) Measurement of the outcome: given that the Urticaria Activity Score (UAS) was incorporated into national guidelines in China in 2019 (Centre for Urticaria Research et al., 2019), outcome measurement methods used before 2019 that followed urticaria-related guidelines and were consistent with the SSRI criteria were considered sufficiently reliable. No study reported differential outcome measurement practices between groups. (5) Selection of the reported result: only three trials documented an ethics approval number, and merely one reported a clinical trial registration number (Liu, 2016; Yang et al., 2018; Wang et al., 2019; Zhan et al., 2024). The predominant failure to specify whether the statistical analysis methods aligned with pre-unblinding protocols, combined with insufficient reporting of detailed specifications for multiple outcomes measures, precludes conclusive evaluation of the selective outcome reporting bias. Regarding TCM-specific criteria, 15 RCTs explicitly described the TCM syndrome associated with the interventions. Among the included RCTs on TCM formulas, the highest-quality studies analyzed *Xiaofeng powder*, *Danggui Yinzi*, and *Yupingfeng powder*. We then provided the RoB quality-adjusted analysis. One study (Liu, 2016) with high risk of bias in three domains was excluded from the data analysis. The results of the bias analysis and quality assessment scoring are presented in Figure 2 and Supplementary Figure S2. Supplementary Appendix Table S3 contains the complete report of RoB 2.0 evaluation.

According to the CINeMA assessment, a total of 39 comparisons were evaluated for binary outcomes, with predominantly moderate overall certainty. High-certainty evidence mainly involved comparisons such as loratadine combined with *Danggui Yinzi*, loratadine combined with *Guizhi decoction,* loratadine combined with *Xiaofeng powder*, ebastine combined with *Yupingfeng powder*, ebastine combined with *Mahuang Fuzi Xixin decoction,* and ebastine combined with *Maxing Shigan decoction*. Comparisons related to *Danggui Yinzi, Yupingfeng powder,* and *Guizhi decoction* generally reached moderate or higher levels of certainty of evidence (Supplementary Appendix Table S4).

Network consistency was assessed using the design-by-treatment interaction model. No significant inconsistency was detected for any outcome (between-designs p-values: SSRI ≥90% p = 0.34; SSRI ≥60% p = 0.52; AEs p = 0.26), indicating agreement between direct and indirect evidence (Supplementary Material S3). Both frequentist and Bayesian consistency assessments provided strong evidence supporting network validity. No meaningful inconsistency was detected (all ΔDIC <2) (Supplementary Figure S3).

For homogeneity, our analysis showed that for SSRI ≥60%, moderate within-design heterogeneity was observed (p = 0.048), primarily in the cetirizine *versus* loratadine comparisons (p = 0.041), suggesting some variability across studies involving this comparison. We employed random-effects models to account for this potential heterogeneity. However, it is notable that the cetirizine *versus* loratadine comparison was not the focus of our analysis. Other outcomes, such as SSRI ≥90% and AEs, fully supported homogeneity (Supplementary Material S3). The funnel plots showed symmetrical distributions (Egger’s test p > 0.10 for all outcomes), suggesting low risk of publication bias (Supplementary Figure S4).

### 3.3 Efficacy evaluation

For the primary outcome (clinical improvements), 36 studies with a total of 3,884 patients were analyzed (Bai, 2011; Cai, 2016; Cao, 2019; 2015; Chen and He, 2013; Cheng, 2016; Fu et al., 2015; Fu and Liu, 2020; Hu et al., 2016; Huang and Duan, 2018; Jiang, 2014; Li et al., 2018; 2006; Lin et al., 2018; Liu, 2020; Niu, 2015; Ou et al., 2005; Shi et al., 2013; Tian and Huang, 2015; Wang and Zhang, 2019; 2018; Wang et al., 2023; 2019; Wu, 2019; Xie et al., 2010; Xie, 2011; Xu, 2024; 2016; Xu et al., 2019; Yin et al., 2003; Yu and Shang, 2016; Zhan et al., 2024; Zhang and Lin, 2011; Zhang, 2023; Zhao, 2015; Zhou, 2020).

**FIGURE 3:**
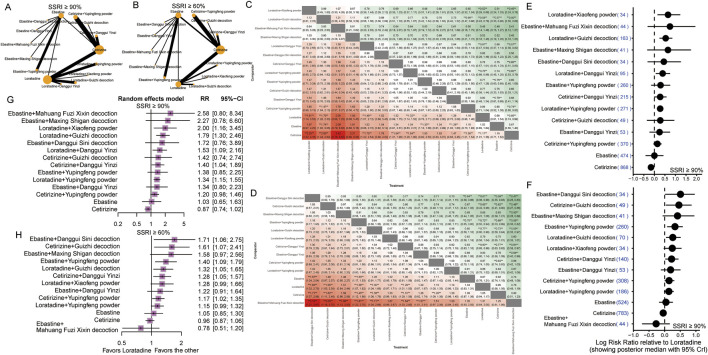
Network meta-analysis measures for clinical improvements. **(A)** Network diagram illustrating the relationships between the treatments for SSRI ≥90%. Nodes represent different interventions (three second-generation antihistamines combined with various TCM formulas), and edges represent direct comparisons in the included trials. The node size is proportional to the number of studies examining each treatment. The edge thickness is proportional to the number of pairwise comparisons. **(B)** Network diagram illustrating the relationships between the treatments for SSRI ≥60%. **(C)** Network estimates for SSRI ≥90% are presented as RR with 95% CrIs. *Statistically significant. **(D)** Network estimates for SSRI ≥60% are presented as RR with 95% CrIs. *Statistically significant. **(E)** Forest plot of the Bayesian model for SSRI ≥90% shows the log RR relative to loratadine. CrI indicates credible interval. **(F)** Forest plot of the Bayesian model for SSRI ≥60% shows the log RR relative to loratadine. CrI indicates credible interval. **(G)** Forest plot of frequentist estimates for SSRI ≥90% shows the RR relative to loratadine. CI indicates confidence interval. **(H)** Forest plot of frequentist estimates for SSRI ≥60% shows the RR relative to loratadine. CI indicates confidence interval.

Bayesian NMA revealed that several combination therapies significantly outperformed monotherapy in achieving SSRI ≥90% response (Figures 3A,C,E,G). Notably, the most effective interventions compared with the corresponding sgAH monotherapies included loratadine combined with *Xiaofeng powder* (RR: 1.91; 95% CrI: 1.14–3.53), loratadine combined with *Guizhi decoction* (RR: 1.70; 95% CrI: 1.28–2.33), loratadine combined with *Danggui Yinzi* (RR: 1.49; 95% CrI: 1.07–2.12), loratadine combined with *Yupingfeng powder* (RR: 1.32; 95% CrI: 1.14–1.52), ebastine combined with *Yupingfeng powder* (RR: 1.33; 95% CrI: 1.13–1.58), ebastine combined with *Danggui Yinzi* (RR: 1.28; 95% CrI: 1.02–1.63), cetirizine combined with *Danggui Yinzi* (RR: 1.54; 95% CrI: 1.21–1.98), and cetirizine combined with *Yupingfeng powder* (RR: 1.36; 95% CrI: 1.21–1.54) (Figure 3C). These findings were further supported by frequentist NMA (Figure 3G).

For the SSRI ≥60% outcome, the network estimates revealed numerous statistically significant pairwise comparisons (Figures 3B,D,F,H). The top-performing interventions included ebastine combined with *Danggui Sini decoction* (RR: 1.57; 95% CrI: 1.04–2.54), ebastine combined with *Yupingfeng powder* (RR: 1.33; 95% CrI: 1.17–1.53), loratadine combined with *Guizhi decoction* (RR: 1.30; 95% CrI: 1.04–1.68), cetirizine combined with *Guizhi decoction* (RR: 1.62; 95% CrI: 1.11–2.46), cetirizine combined with *Danggui Yinzi* (RR: 1.31; 95% CrI: 1.09–1.58), and cetirizine combined with *Yupingfeng powder* (RR: 1.20; 95% CrI: 1.09–1.36) (Figure 3D). All these combinations achieved statistical significance when compared with the corresponding sgAH monotherapies.

**FIGURE 4:**
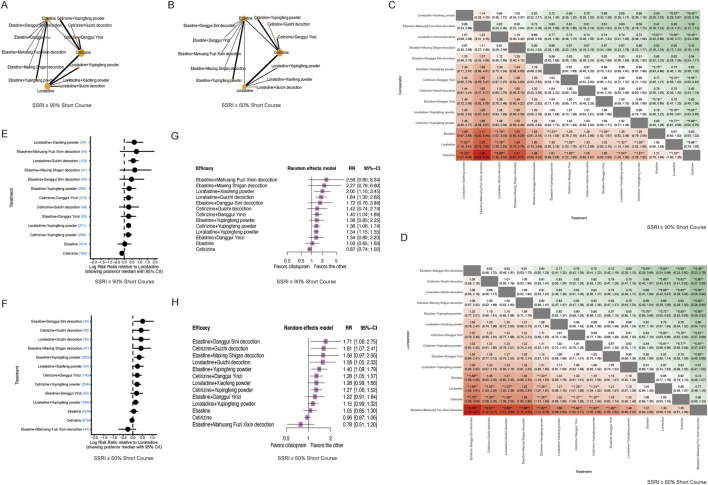
Network meta-analysis of clinical improvements in the short-course treatment subgroup. **(A)** Network diagram illustrating the relationships between treatments for the SSRI ≥90% short-course subgroup. Nodes represent different interventions (second-generation antihistamines alone or combined with TCM formulas), and edges represent direct comparisons in the included trials. **(B)** Network diagram illustrating the relationships between treatments for the SSRI ≥60% short-course subgroup. **(C)** Network estimates for the SSRI ≥90% short-course subgroup are presented as RR with 95% CrIs. * Statistically significant. **(D)** Network estimates for the SSRI ≥60% short-course subgroup are presented as RR with 95% CrIs. * Statistically significant. **(E)** Forest plot of the Bayesian model for the SSRI ≥90% short-course subgroup shows the log RR relative to loratadine. The plot displays the posterior median with 95% CrIs. **(F)** Forest plot of the Bayesian model for the SSRI ≥60% short-course subgroup shows the log RR relative to loratadine. The plot displays the posterior median with 95% CrIs. **(G)** Forest plot of frequentist estimates for the SSRI ≥90% short-course subgroup shows the RR relative to loratadine. CI indicates confidence interval. **(H)** Forest plot of frequentist estimates for the SSRI ≥60% short-course subgroup shows the RR relative to loratadine. CI indicates confidence interval.

We subdivided the duration of the treatment course into short course (≤5 weeks) and long course (>5 weeks). In the short-course subgroup, most TCM combinations with sgAHs showed higher effect estimates than sgAH monotherapy (Figure 4). For the SSRI ≥90% outcome, statistically significant benefits were observed for loratadine combined with *Xiaofeng powder* (RR: 1.92; 95% CrI: 1.15–3.41), loratadine combined with *Guizhi decoction* (RR: 1.79; 95% CrI: 1.29–2.53), loratadine combined with *Yupingfeng powder* (RR: 1.32; 95% CrI: 1.15–1.54), cetirizine combined with *Danggui Yinzi* (RR: 1.55; 95% CrI: 1.22–1.99), and ebastine combined with *Yupingfeng powder* (RR: 1.33; 95% CrI: 1.14–1.58) (Figures 4C,E,G). In the short-course subgroup of the SSRI ≥60% outcome, the statistical significance of combination regimens was more consistent. Ebastine combined with *Danggui Sini decoction* (RR: 1.58; 95% CrI: 1.05–2.65), ebastine combined with *Yupingfeng powder* (RR: 1.33; 95% CrI: 1.16–1.53), cetirizine combined with *Guizhi decoction* (RR: 1.59; 95% CrI: 1.09–2.42), cetirizine combined with *Danggui Yinzi* (RR: 1.31; 95% CrI: 1.09–1.60), cetirizine combined with *Yupingfeng powder* (RR: 1.30; 95% CrI: 1.12–1.54), and loratadine combined with *Guizhi decoction* (RR: 1.53, 95% CrI: 1.03–2.39) all showed statistically significant superiority over the corresponding monotherapies, with relatively narrower credible intervals. Ebastine combined with *Maxing Shigan decoction,* ebastine combined with *Danggui Yinzi,* cetirizine combined with *Danggui Yinzi,* loratadine combined with *Xiaofeng powder,* and loratadine combined with *Yupingfeng powder* showed consistent benefit, but it did not reach statistical significance (Figures 4D,F,H).

**FIGURE 5:**
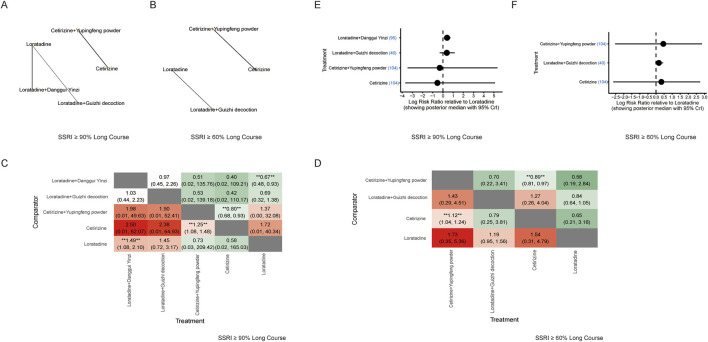
Network meta-analysis of clinical improvements in the long-course treatment subgroup. **(A)** Network diagram illustrating the relationships between treatments for the SSRI ≥90% long-course subgroup. Nodes represent different interventions, and edges represent direct comparisons in the included trials. **(B)** Network diagram illustrating the relationships between the treatments for the SSRI ≥60% long-course subgroup. **(C)** Network estimates for SSRI ≥90% (long-course) are presented as RR with 95% CrIs. * Statistically significant. **(D)** Network estimates for SSRI ≥60% (long-course) are presented as RR with 95% CrIs. * Statistically significant. **(E)** Forest plot of the Bayesian model for the SSRI ≥90% long-course subgroup shows the log RR relative to loratadine. CrI indicates credible interval. **(F)** Forest plot of the Bayesian model for the SSRI ≥60% long-course subgroup shows the log RR relative to loratadine. CrI indicates credible interval.

In the long-course subgroup, for studies with SSRI ≥90%, cetirizine combined with *Yupingfeng powder* was significantly superior to cetirizine alone (RR: 1.25; 95% CrI: 1.08–1.48), and loratadine combined with *Danggui Yinzi* was superior to loratadine monotherapy (RR: 1.49; 95% CrI: 1.08–2.10). Loratadine combined with *Guizhi decoction* showed superior efficacy than loratadine monotherapy (Figures 5C,E). For SSRI ≥60%, the analytical results similarly supported the superiority of combined therapy, with cetirizine combined with *Yupingfeng powder* being significantly more effective than cetirizine alone (RR: 1.12; 95% CrI: 1.04–1.24). Greater efficacy was observed for loratadine combined with *Guizhi decoction versus* loratadine monotherapy (Figures 5D,F).

### 3.4 Recurrence evaluation

**FIGURE 6:**
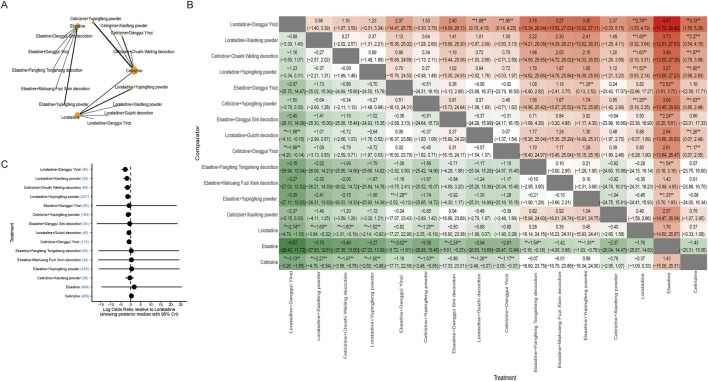
Network meta-analysis of recurrence rates for different interventions. **(A)** Network diagram illustrating the relationships between treatments for recurrence rates. Nodes represent different interventions, and edges represent direct comparisons in the included trials. The node size is proportional to the total number of patients, and the edge thickness is proportional to the number of studies. **(B)** Network estimates for recurrence rates are presented as log OR with 95% CrIs. *Statistically significant. **(C)** The forest plot of the Bayesian model shows the log OR relative to loratadine. The plot displays the posterior median with 95% CrIs.

The recurrence rate was analyzed as a secondary outcome in this NMA. The analysis included 24 studies with a total of 2,465 patients involved in two disconnected networks (Figure 6A). Among the treatments evaluated, the treatments combining antihistamines (particularly loratadine and ebastine) with *Danggui Yinzi* demonstrated the highest probability of achieving superior rankings (with smaller recurrence rates) (Figure 6B). For recurrence prevention specifically, loratadine combined with *Danggui Yinzi* demonstrated superior efficacy than at least six other treatments, while cetirizine monotherapy appeared to be the least effective option for preventing recurrence (Figure 6C). Compared with loratadine monotherapy, better recurrence prevention was seen with loratadine combined with *Danggui Yinzi* (log OR: −2.74; 95% CrI: −4.79 to −1.33), loratadine combined with *Xiaofeng powder* (log OR: −1.89; 95% CrI: −3.64 to −0.32), loratadine combined with *Yupingfeng powder* (log OR: −1.52; 95% CrI: −2.14 to −0.93), ebastine combined with *Danggui Yinzi* (log OR: −2.62; 95% CrI: −3.72 to −1.61), ebastine combined with *Danggui Sini decoction* (log OR: −2.24; 95% CrI: −5.61 to −0.25), ebastine combined with *Fangfeng Tongsheng decoction* (log OR: −1.54; 95% CrI: −3.16 to −0.18), ebastine combined with *Yupingfeng powder* (log OR: −1.33; 95% CrI: −1.93 to −0.76), cetirizine combined with *Chushi Weiling decoction* (log OR: −1.97; 95% CrI: −3.56 to −0.76), cetirizine combined with *Yupingfeng powder* (log OR: −1.63; 95% CrI: −2.48 to −0.85), and cetirizine combined with *Danggui Yinzi* (log OR: −1.17; 95% CrI: −2.05 to −0.37) (Figure 6C).

In the long-course subgroup (>12 weeks of follow-up), combination therapies demonstrated superior efficacy than monotherapy. Both ebastine combined with *Danggui Yinzi* (log OR: −2.61; 95% CrI: −3.73 to −1.62) and loratadine combined with *Yupingfeng powder* (log OR: −2.84; 95% CrI: −6.23 to −1.01) outperformed their respective monotherapies significantly (Supplementary Figure S5A,C,E).

In the short-course subgroup (≤12 weeks of follow-up), combining sgAH with TCM (e.g., *Danggui Yinzi or Yupingfeng powder*) reduced the recurrence risk more significantly than monotherapy alone (Supplementary Figure S5B,D,F).

### 3.5 Safety evaluation

**FIGURE 7:**
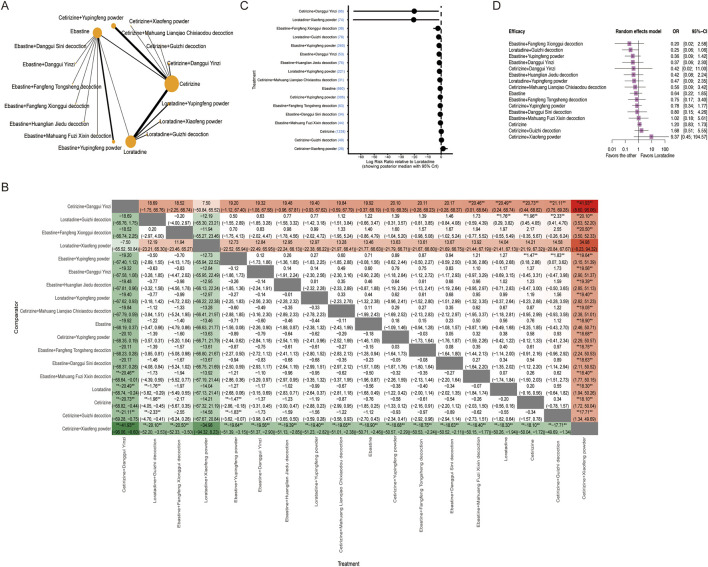
Network meta-analysis of AEs for different interventions. **(A)** Network diagram illustrating the relationships between treatments for AEs. Nodes represent different interventions (second-generation antihistamines alone or combined with TCM formulas), and edges represent direct comparisons in the included trials. The node size is proportional to the total number of patients, and the edge thickness is proportional to the number of studies. **(B)** Network estimates for AEs are presented as log OR with 95% CrIs. *Statistically significant. **(C)** The forest plot of the Bayesian model shows the log OR relative to loratadine. The plot displays the posterior median with 95% CrIs. **(D)** The forest plot of frequentist estimates shows the OR relative to loratadine. CI indicates confidence interval.

A total of 31 studies with 3,930 patients were included in the safety network analysis (Figure 7A). According to the league table, cetirizine combined with Xiaofeng powder appeared to be associated with a higher incidence of adverse events than several other treatments (Figure 7B). Compared with cetirizine monotherapy, three combination therapies demonstrated significantly lower AE rates: cetirizine combined with *Danggui Yinzi* (log OR: −20.73; 95% CrI: −68.82 to −0.44), loratadine combined with *Guizhi decoction* (log OR: −1.96; 95% CrI: −4.05 to −0.45), and ebastine combined with *Yupingfeng powder* (log OR: −1.47; 95% CrI: −2.86 to −0.18). In the Bayesian NMA, loratadine combined with *Guizhi decoction* demonstrated a statistically significant effect (log OR: −1.96; 95% CrI: 4.05 to −0.45), indicating a high probability of true clinical benefit. In contrast, the frequentist analysis yielded a nonsignificant estimate (OR: 0.20; 95% CrI: 0.06–1.06), with a confidence interval narrowly crossing the null. Given the Bayesian framework’s ability to directly quantify the probability of effect and incorporate prior information, the Bayesian results are considered more reliable for clinical interpretation (Figures 7C,D).

We categorized AEs according to the Common Terminology Criteria for Adverse Events (CTCAE) version 5.0. For grade 1 (mild) AEs (Supplementary Appendix Table S5; Supplementary Figure S6), monotherapy was associated with higher risks than combination therapy, particularly for cetirizine *versus* cetirizine combined with *Mahuang Lianqiao Chixiaodou decoction* (log OR: −16.75; 95% CrI: −56.01 to −0.71) and ebastine *versus* ebastine combined with *Fangfeng Xionggui decoction* (log OR: −16.78; 95% CrI: −56.22 to −0.21). For grade 2 (moderate) AEs, loratadine combined with *Guizhi decoction* (log OR: −2.16; 95% CrI: −5.46 to −0.18) and cetirizine combined with *Danggui Yinzi* (log OR: −23.09; 95% CrI: −76.14 to −0.50) significantly reduced the risk of moderate AEs compared to that with monotherapies. These findings indicate that the evaluated TCM formula–antihistamine combinations may have a more favorable safety profile than antihistamine monotherapy.

### 3.6 Laboratory measures

We also compared different treatment regimens by evaluating their effects on IgE (Supplementary Figures S7A,B) and IL-4 (Supplementary Figures S7C,D). The Bayesian meta-analysis revealed that none of the evaluated treatments demonstrated a statistically significant superiority over cetirizine in reducing IgE or IL-4 levels. While combination therapies (e.g., cetirizine with traditional powders/decoctions) and other antihistamines (e.g., ebastine and loratadine) were assessed, their effects on both the biomarkers were not significantly different from that of cetirizine alone.

### 3.7 Sensitivity analysis

To evaluate the impact of intervention heterogeneity arising from syndrome-based modifications of TCM prescriptions on study outcomes, we performed a sensitivity analysis. Prescriptions containing ≤5 herbs were excluded if the proportion of the modified herbs exceeded 50%, while those containing >5 herbs were excluded if the proportion exceeded 40%. In the SSRI ≥90% endpoint (Supplementary Figures S8), combinations including loratadine combined with *Guizhi decoction,* loratadine combined with *Danggui Yinzi*, cetirizine combined with *Danggui Yinzi*, loratadine combined with *Yupingfeng powder*, cetirizine combined with *Yupingfeng powder,* and ebastine combined with *Yupingfeng powder* were significantly superior to their respective monotherapies in both the primary and sensitivity analyses. In contrast, the benefit associated with cetirizine combined with *Guizhi decoction* remained nonsignificant despite a positive trend (Supplementary Figures S8A,B). In the SSRI ≥60% outcome, cetirizine combined with *Guizhi decoction*, cetirizine combined with *Danggui Yinzi,* cetirizine combined with *Yupingfeng powder*, ebastine combined with *Yupingfeng powder*, and ebastine combined with *Danggui Sini decoction* all showed significant benefit in both the primary analysis and sensitivity analysis; ebastine combined with *Danggui Yinzi* showed consistent benefit in both analyses but did not reach statistical significance (Supplementary Figures S8C,D).

We conducted sensitivity analyses excluding the studies with high risk of bias in at least two domains (Li, 2021; Li, 2021; Lu, 2011; Wu and Lan, 2023; Ying, 2022; Yang et al., 2024; Yang et al., 2021; Mei et al., 2024; Mao LD, 2023, Zhuang, 2005; Lin and Li, 2009). An important observation from this re-evaluation is that none of the studies with two high-risk domains were actually included in the SSRI-based primary outcome analysis. These studies either (1) reported different outcome measures that could not be harmonized to the SSRI or (2) were excluded during data screening for other reasons. Consequently, the sensitivity analysis results are consistent with the primary analysis results, which strengthens our confidence in the findings.

## 4 Discussion

This study included 50 RCTs and applied both Bayesian and frequentist network meta-analyses to systematically compare the efficacy, recurrence, and safety of classical TCM formulas combined with sgAHs for CSU and establish a ranking of the combination regimens. The results showed that most classical formula–sgAH combinations were superior to antihistamine monotherapy for both the SSRI ≥90% and SSRI ≥60% outcomes. Among them, regimens involving *Danggui Yinzi, Yupingfeng powder*, and *Guizhi decoction* ranked highly in terms of clinical efficacy and recurrence prevention, indicating that integrated TCM–antihistamine therapy may provide advantages in symptom control and long-term disease management.

In both the long-course and short-course subgroups, loratadine combined with classical TCM formulas was superior to loratadine monotherapy. Among these, the combination of loratadine and *Guizhi decoction* demonstrated superior performance in both treatment durations. As a chronic relapsing condition, the therapeutic goal of CSU extends beyond short-term symptom control to sustained remission and prevention of recurrence. Our findings suggest that certain classical TCM formulas may exert sustained regulatory effects on disease activity, offering a potential alternative strategy for patients who are unable to tolerate long-term dose escalation or biologic therapies. In terms of safety, most combination regimens did not increase the incidence of AEs. Notably, some combinations, such as loratadine combined with *Guizhi decoction* and cetirizine combined with *Danggui Yinzi*, were associated with a lower risk of AEs. The remaining combinations showed no significant increase in AE risk. According to the CTCAE (version 5.0) grading analysis, mild-to-moderate AEs were generally manageable in the combination groups, supporting the favorable tolerability of classical TCM formulas when administered alongside standard-dose antihistamines.

Notably, in the network analyses of serum IgE and IL-4 levels, none of the combination regimens demonstrated a statistically significant advantage over sgAH monotherapy. These findings indicate that clinical improvement may not necessarily translate into short-term reductions in circulating immunological marker levels, and it may also reflect limitations related to small sample sizes and variability in assessment time points. Therefore, future studies incorporating standardized immunological endpoints are warranted to further elucidate the underlying biological mechanisms.

Mast cells are the priming cells in CSU. Their degranulation releases vasoactive mediators such as histamine, which activate sensory nerve endings, causing vasodilation and increased vascular permeability, resulting in wheals and pruritus (Kaplan et al., 2023). sgAHs rapidly suppress histamine-mediated vasodilation and nerve sensitization by competitively blocking H1 receptors, thereby quickly relieving acute symptoms. However, histamine is not the only inflammatory mediator in CSU (Kaplan et al., 2023). The essence of the disease lies in a broader imbalance in the immune–inflammatory network (Kaplan et al., 2023; Wang and Li, 2023; Kolkhir et al., 2024). Although antihistamines can block histamine signaling, they struggle to reverse this state of immune imbalance and cannot sustainably inhibit mast cell activation. This is precisely the key mechanism underlying their inadequate long-term efficacy (Kaplan et al., 2023). Classical TCM formulas may enhance the therapeutic efficacy of sgAH in the treatment of CSU through multipathway and multitarget mechanisms (Zhang et al., 2014; 2024). Our study findings suggest that specific combinations, such as *Danggui Yinzi* combined with loratadine, demonstrate particular clinical benefits in the treatment of CSU.

In the current field of clinical research on TCM, especially in early studies, widespread methodological flaws and lack of reporting standards remain a major concern. Merging analyses based solely on pooled data without rigorously assessing the methodological robustness of the original studies may introduce significant bias, leading to conclusions that deviate from clinical practice. These flaws may lead to overestimation of the therapeutic effects for certain Chinese medicine interventions. In our study, some included studies had methodological weaknesses, including limited blinding (most were open-label trials), unclear allocation concealment, and relatively small sample sizes. These limitations have been formally assessed using the Cochrane RoB 2.0 tool, with the majority of studies rated as having “some concerns” or “high risk” of bias, which has been incorporated into our interpretation of the evidence quality through the CINeMA framework. In addition, the network includes only 22 direct comparisons out of 230 possible pairwise comparisons, resulting in a sparse network. This sparsity increases reliance on indirect evidence, which is subject to the transitivity assumption. We have conducted consistency checks to demonstrate that the transitivity assumption is plausible. Future large-scale, well-designed RCTs are needed to confirm our findings before widespread clinical implementation can be recommended.

In summary, this study provides a systematic ranking of classical TCM formulas combined with sgAHs for the treatment of CSU, supporting the potential value of specific combination regimens in enhancing the efficacy and reducing recurrence. High-quality head-to-head RCTs with long-term follow-up are still needed to further clarify their clinical positioning.

## 5 Conclusion

This NMA systematically compared the relative efficacy and safety of classical TCM formulas combined with sgAHs for CSU for the first time. The findings indicate that regimens involving *Danggui Yinzi, Yupingfeng powder,* and *Guizhi decoction* demonstrated superior performance in symptom improvement and recurrence prevention without increasing the risk of AEs. Overall, this study provides evidence-based support for the integration of these classical formulas into CSU management.

## Data Availability

The raw data supporting the conclusions of this article will be made available by the authors, without undue reservation.

## References

[B1] Bai W. (2011). Clinical observation of Xiaofeng powder with modifications combined with loratadine in the treatment of chronic urticaria. Zhonguo Zhong Xi Yi Jie He Pi Fu Xing Bing Xue Za Zhi 10, 300–301. 10.3969/j.issn.1672-0709.2011.05.013

[B2] Cai X. (2016). Observation on the efficacy of Yupingfeng granules combined with ebastine in the treatment of chronic idiopathic urticaria. Lin. Chuang He Li Yong Yao 9, 78–79. 10.15887/j.cnki.13-1389/r.2016.28.049

[B3] Cao Y. (2015). Loratadine combined with Guizhi decoction in the treatment of chronic urticaria for 40 cases. Zhongguo Zhong Yi Yao Xian Dai Yuan Cheng Jiao Yu 13, 70–71. 10.3969/j.issn.1672-2779.2015.18.036

[B4] Cao H. (2019). Efficacy of modified Danggui Yin in treating Chronic Urticaria (CU) and its impact on immune indicators and quality of life in patients. Neimenggu Zhong Yi Yao 38, 6–7. 10.16040/j.cnki.cn15-1101.2019.10.004

[B5] Centre for Urticaria Research, Chinese Society of Dermatology, Xu J. Hao F. Tang H. (2019). Guideline for diagnosis and treatment of urticaria in China (2018). Chin. J. Dermatol. 52, 1–5. 10.3760/cma.j.issn.0412-4030.2019.01.001

[B6] Centre for Urticaria Research, Chinese Society of Dermatology, Xu J. Hao F. Tang H. Zhao Z. Yao X. (2022). Guideline for diagnosis and treatment of urticaria in China (2022). Chin. J. Dermatol. 55, 1041–1049. 10.3760/cjd.20220609

[B7] Chen Y. He W. (2013). Analysis of the therapeutic efficacy of ebastine and yupingfeng in treatment of chronic urticaria. Zhongguo Dang Dai Yi Yao 20, 85–86.

[B8] Chen J. Zhou M. Liu X. Li Y. Jian Q. Zhang X. (2012). Clinical research on Chinese medicine for chronic urticaria: a systematic review. Shanghai J. Tradit. Chin. Med. 46, 57–60. 10.16305/j.1007-1334.2012.09.027

[B9] Chen S. Cao W. Xiao X. Wang L. Wan R. Zou Z. (2024). A systematic review and meta-analysis of efficacy and safety of compound glycyrrhizin combined with second-generation non-sedated antihistamine for the treatment of chronic urticaria. J. Dermatol. Treat. 35, 2299597. 10.1080/09546634.2023.2299597 38166511

[B10] Cheng J. (2016). Clinical Study on danggui yinzi combined with Western medicine in the treatment of chronic urticaria with blood deficiency and wind-dryness syndrome. Xin Zhong Yi 48, 184–186. 10.13457/j.cnki.jncm.2016.04.070

[B11] Fricke J. Ávila G. Keller T. Weller K. Lau S. Maurer M. (2020). Prevalence of chronic urticaria in children and adults across the globe: systematic review with meta-analysis. Allergy 75, 423–432. 10.1111/all.14037 31494963

[B12] Fu J. Liu C. (2020). Clinical observation of 80 cases of chronic urticaria treated with levocetirizine combined with modified Yupingfeng powder. Hunan Zhong Yi Za Zhi 36, 54–55. 10.16808/j.cnki.issn1003-7705.2020.03.021

[B13] Fu H. Zhang Z. Xu M. (2015). The efficacy of Yupingfeng granules as an adjuvant therapy for chronic urticaria of blood deficiency and wind dryness type. Xin Zhong Yi 47, 174–176. 10.13457/j.cnki.jncm.2015.07.078

[B14] Gammeri L. Panzera C. Calapai F. Cicero N. Gangemi S. (2023). Asian herbal medicine and chronic urticaria: which are the therapeutic perspectives? Nat. Prod. Res. 37, 1917–1934. 10.1080/14786419.2022.2122055 36094856

[B15] Gonçalo M. Gimenéz‐Arnau A. Al‐Ahmad M. Ben‐Shoshan M. Bernstein J. A. Ensina L. F. (2021). The global burden of chronic urticaria for the patient and society. Br. J. Dermatol. 184, 226–236. 10.1111/bjd.19561 32956489

[B16] Guillén‐Aguinaga S. Jáuregui Presa I. Aguinaga‐Ontoso E. Guillén‐Grima F. Ferrer M. (2016). Updosing nonsedating antihistamines in patients with chronic spontaneous urticaria: a systematic review and meta‐analysis. Br. J. Dermatol. 175, 1153–1165. 10.1111/bjd.14768 27237730

[B17] Heinrich M. Jalil B. Abdel-Tawab M. Echeverria J. Kulić Ž. McGaw L. J. (2022). Best Practice in the chemical characterisation of extracts used in pharmacological and toxicological research-The ConPhyMP-Guidelines. Front. Pharmacol. 13, 953205. 10.3389/fphar.2022.953205 36176427 PMC9514875

[B18] Hu Y. Li L. Ren T. Li L. (2016). Efficacy of modified Danggui Decoction in treating chronic urticaria and its impact on immune indicators and quality of life. Shanxi Zhong Yi 37, 85–86.

[B19] Huang Y. Duan Q. (2018). Effects of modified danggui decoction combined with ebastine on T lymphocyte subsets, serum total IgE and diamine oxidase activity in patients with chronic urticaria. Zhonguo Yao Shi 21, 2163–2165. 10.3969/j.issn.1008-049X.2018.12.018

[B20] Hutton B. Salanti G. Caldwell D. M. Chaimani A. Schmid C. H. Cameron C. (2015). The PRISMA extension statement for reporting of systematic reviews incorporating network meta-analyses of health care interventions: checklist and explanations. Ann. Intern. Med. 162, 777–784. 10.7326/M14-2385 26030634

[B21] Jiang J. (2014). Observation on the efficacy of ebastine tablets combined with Yupingfeng capsules in the treatment of chronic urticaria. Heilongjiang Yi Yao 27, 1122–1123. 10.14035/j.cnki.hljyy.2014.05.208

[B22] Kaplan A. Lebwohl M. Giménez-Arnau A. M. Hide M. Armstrong A. W. Maurer M. (2023). Chronic spontaneous urticaria: focus on pathophysiology to unlock treatment advances. Allergy 78, 389–401. 10.1111/all.15603 36448493

[B23] Kolkhir P. Bonnekoh H. Metz M. Maurer M. (2024). Chronic spontaneous urticaria: a review. JAMA 332, 1464–1477. 10.1001/jama.2024.15568 39325444

[B24] Li G. (2021). Observation on the efficacy of modified Guizhi Decoction combined with ebastine tablets in the treatment of chronic urticaria. Shi Yong Zhong Yi Yao Za Zhi 37, 1694–1695.

[B25] Li L. Ma T. (2021). Efficacy analysis of modified Yupingfeng powder combined with ebastine in the treatment of chronic urticaria. Zhongguo Shi Yong Yi Yao 16, 178–180. 10.14163/j.cnki.11-5547/r.2021.25.068

[B26] Li Z. Chen Y. Tian C. (2006). Clinical research of desloradine in the treatment of chronic urticaria. Pi Fu Bing Yu Xing Bing, 28 (1), 11–12.

[B27] Li Y. Yang F. Li H. (2018). Clinical study on Yupingfeng granules combined with levocetirizine capsules in the treatment of chronic urticaria with superficies deficiency with poor consolidation. Shi Jie Zui Xin Yi Xue Xin Xi Wen Zhai 18, 146–147. 10.19613/j.cnki.1671-3141.2018.85.111

[B28] Lin W. Xue X. Xue W. Lin Y. (2018). Efficacy and safety of desloratadine in the treatment of chronic urticaria. Pi Fu Xing Bing Zhen Liao Xue Za Zhi 25, 350–353.

[B70] Lin Y. Li Y. (2009). “Comparison of the efficacy of levocetirizine hydrochloride and loratadine in the treatment of chronic urticaria,” in Proceedings of the 4th National Academic Conference on Integrative Medicine in Allergy. (Xi’an, Shanxi). 2, 230–231. Available online at: https://kns.cnki.net/KCMS/detail/detail.aspx?dbcode=CPFD&dbname=CPFD0914&filename=ZGZP200904001083 (Accessed February 3, 2025).

[B29] Liu N. (2016). Evaluation of the efficacy of traditional Chinese medicine danggui yinzi combined with cetirizine hydrochloride in the treatment of chronic urticaria. Shi Jie Zui Xin Yi Xue Xin Xi Wen Zhai 16, 112–113.

[B30] Liu X. (2020). Comparison of the effects of ebastine and cetirizine on clinical symptom scores and adverse reactions in patients with chronic urticaria. Pi Fu Bing Yu Xing Bing 42, 854–856.

[B31] Lu Y. (2011). Treatment of 40 cases of wind-heat type chronic urticaria with modified Xiaofeng powder combined with loratadine. Henan Zhong Yi 31, 913–914. 10.16367/j.issn.1003-5028.2011.08.085

[B32] Mao L. (2023). Efficacy observation of modified Chushi Weiling Decoction combined with levocetirizine hydrochloride tablets in treating chronic urticaria of spleen deficiency type. Zhongguo Xian Dai Yao Wu Ying Yong 17, 163–165. 10.14164/j.cnki.cn11-5581/r.2023.18.045

[B33] Maurer M. Abuzakouk M. Bérard F. Canonica W. Oude Elberink H. Giménez‐Arnau A. (2017). The burden of chronic spontaneous urticaria is substantial: real‐world evidence from ASSURE‐CSU. Allergy 72, 2005–2016. 10.1111/all.13209 28543019 PMC5724512

[B34] Mei C. Lu D. Chen X. (2024). Clinical Study on modified Mahuang Lianqiao Chixiaodou decoction combined with levocetirizine hydrochloride tablets for chronic urticaria. Xin Zhong Yi 56, 13–17. 10.13457/j.cnki.jncm.2024.07.003

[B35] Nikolakopoulou A. Higgins J. P. T. Papakonstantinou T. Chaimani A. Del Giovane C. Egger M. (2020). CINeMA: an approach for assessing confidence in the results of a network meta-analysis. PLoS Med. 17, e1003082. 10.1371/journal.pmed.1003082 32243458 PMC7122720

[B36] Niu Y. (2015). Clinical observation of 64 cases of chronic urticaria treated with Yupingfeng powder combined with levocetirizine. Pi Fu Bing Yu Xing Bing 37, 300–301.

[B37] Ou B. Hao S. Liu W. Wang J. Jing L. (2005). Comparison of the efficacy of levocetirizine and loratadine in the treatment of chronic urticaria. Zhongguo Ma Feng Pi Fu Bing Za Zhi. 10 (10), 75–76.

[B38] Papakonstantinou T. Nikolakopoulou A. Higgins J. P. T. Egger M. Salanti G. (2020). CINeMA: software for semiautomated assessment of the confidence in the results of network meta-analysis. Campbell Syst. Rev. 16, e1080. 10.1002/cl2.1080 37131978 PMC8356302

[B71] Potter P. C. Kapp A. Maurer M. Guillet G. Jian A. M. Hauptmann B. (2009). Comparison of the efficacy of levocetirizine 5 mg and desloratadine 5 mg in chronic idiopathic urticaria patients. Allergy 64, 596–604. 10.1111/j.1398-9995.2008.01893.x 19053988

[B39] Sharma M. Bennett C. Cohen S. N. Carter B. (2014). H1-antihistamines for chronic spontaneous urticaria. Cochrane Database Syst. Rev. 2014, CD006137. 10.1002/14651858.CD006137.pub2 25397904 PMC6481497

[B40] Shi C. Chen K. Wang M. Kuang Q. Zhang D. Mao H. (2013). Effect of Chinese Angelica decoction plus cetirzine hydrochloride in treatment of chronic urticaria. Zhongguo Quan Ke Yi Xue 16, 4102–4105.

[B41] Sterne J. A. C. Savović J. Page M. J. Elbers R. G. Blencowe N. S. Boutron I. (2019). RoB 2: a revised tool for assessing risk of bias in randomised trials. BMJ 366, l4898. 10.1136/bmj.l4898 31462531

[B42] Tian J. Huang S. (2015). Therapeutic observation of the effect of combining therapy of desloratadine citrate disodium capsules and yupingfeng on chronic urticaria and effects on serum levels of IgE. Zhongguo Yi Kan. 50, 79–81.

[B43] Wang J. Li J. (2023). Research progress in the pathogenesis of chronic urticaria. J. Cent. South Univ. Sci. 48, 1602–1610. 10.11817/j.issn.1672-7347.2023.230037 38432889 PMC10929888

[B44] Wang C. Zhang C. (2018). Efficacy of modified Yupingfeng Powder combined with levocetirizine in the treatment of chronic urticaria and its effect on Serum immune inflammatory factors. Xian Dai Zhong Xi Yi Jie He Za Zhi 27, 291–295.

[B45] Wang B. Zhang S. (2019). Clinical effect analysis of modified Yupingfeng Powder combined with desloratadine in the treatment of chronic urticaria. Zhongguo Xian Dai Yao Wu Ying Yong 13, 147–148. 10.14164/j.cnki.cn11-5581/r.2019.16.085

[B46] Wang Y. Weining C. Jun M. Lu J. (2019). Exploring the clinical effect and effect of Yupingfeng granule combined with sedative ralliplatin in the treatment of chronic urticaria. Shi Jie Fu He Yi Xue 5, 120–122.

[B47] Wang L. Han T. Lu J. Li Y. Zhao B. (2023). Effect of danggui Sini decoction combined with ebastine tablets in the treatment of chronic urticaria. Lin. Chuang Yi Xue 43, 122–125. 10.19528/j.issn.1003-3548.2023.06.039

[B48] Wu X. (2019). Efficacy analysis of modified Guizhi decoction combined with conventional Western medicine in the treatment of chronic idiopathic urticaria. Pi Fu Bing Yu Xing Bing 41, 244–245.

[B49] Wu S. Lan C. (2023). Clinical study on the treatment of children’s chronic spontaneous urticaria with blood-heat and wind syndrome by Liangxue Xiaofeng powder combined with levocetirizine oral solution. Shizhen Guo Yi Guo Yao 34, 2168–2171.

[B50] Xie S. (2011). The efficacy of modified Yupingfeng Powder combined with levocetirizine in the treatment of chronic urticaria. Guangxi Zhong Yi Xue Yuan Xue Bao 14, 34–35. 10.3969/j.issn.1008-7486.2011.02.021

[B51] Xie Q. Sun Y. Zhao G. Liu B. (2010). Comparison of the efficacy of four antihistamines in the treatment of chronic urticaria. Zhonguo Ma Feng Pi Fu Bing Za Zhi 26, 195.

[B52] Xu F. (2016). Observation on the efficacy of levocetirizine capsules combined with Yupingfeng granules in the treatment of chronic urticaria. Zhong Xi Yi Jie He Xin Xue Guan Bing Za Zhi 4, 57–60. 10.16282/j.cnki.cn11-9336/r.2016.31.038

[B53] Xu D. (2024). Observation on the effect and adverse reactions of modified Guizhi decoction combined with levocetirizine hydrochloride in the treatment of chronic urticaria. Ying Sheng 9, 17–19.

[B54] Xu J. Wang Y. Wang L. Liu A. Fang Y. (2019). Clinical efficacy and safety of Mahuang Fuzi Xixin Decoction combined with ebastine in the treatment of chronic spontaneous urticaria with spleen deficiency and accumulated Dampness syndrome. Zhonguo Quan Ke Yi Xue 22, 142–145.

[B55] Yang S.-H. Lin Y.-H. Lin J.-R. Chen H.-Y. Hu S. Yang Y.-H. (2018). The efficacy and safety of a fixed combination of Chinese herbal medicine in chronic urticaria: a randomized, double-blind, placebo-controlled pilot study. Front. Pharmacol. 9, 1474. 10.3389/fphar.2018.01474 30618764 PMC6305335

[B56] Yang R. Zhu Q. Huang X. (2021). Observation on the effect of Jiawei Huanglian Jiedu decoction combined with ebastine in the treatment of patients with blood-heat syndrome of chronic urticaria. Baotou Yi Xue Yuan Xue Bao 37, 82–86. 10.16833/j.cnki.jbmc.2021.01.023

[B57] Yang L. Lou D. Li J. Yu G. (2024). Clinical Study on modified Fangfeng Xionggui Decoction combined with ebastine tablets for chronic urticaria with blood deficiency and wind-dryness syndrome. Xin Zhong Yi 56, 63–67. 10.13457/j.cnki.jncm.2024.13.012

[B58] Yin R. Diao Q. Ye Q. (2003). Clinical research of three antihistamines in the treatment of chronic idiopathic urticaria. Lin. Chuang Pi Fu Ke Za Zhi. 32 (11), 675–677.

[B59] Ying Z. (2022). Effect of Fangfeng Tongsheng granules combined with ebastine on patients with chronic urticaria. Zhong Wai Yi Xue Yan Jiu 20, 120–123. 10.14033/j.cnki.cfmr.2022.23.030

[B60] Yu X. Shang P. (2016). Guizhi Decoction Combined with desloratadine citrate clinical observation on the treatment of chronic urticaria. Shi Jie Zui Xin Yi Xue Xin Xi Wen Zhai 16, 146–147.

[B61] Zhan Y. Wang Y. Lv W. (2024). Effect of Danggui Yinzi combined with loratadine in the treatment of chronic urticaria. Zhonguo Xian Dai Yi Sheng 62, 84–87. 10.3969/j.issn.1673-9701.2024.06.019

[B62] Zhang Y. (2023). Effect of modified maxing shigan decoction combined with ebastine tablets on chronic urticaria. Zhong Wai Yi Xue Yan Jiu 21, 17–20. 10.14033/j.cnki.cfmr.2023.11.005

[B63] Zhang T. Lin Q. (2011). Clinical observation of Danggui Decoction combined with levocetirizine hydrochloride in the treatment of chronic urticaria. Zhongguo Zhong Xi Yi Jie He Pi Fu Xing Bing Xue Za Zhi 10, 43–44.

[B64] Zhang A. Sun H. Wang X. (2014). Potentiating therapeutic effects by enhancing synergism based on active constituents from traditional medicine. Phytother. Res. PTR 28, 526–533. 10.1002/ptr.5032 23913598

[B65] Zhang L. Yang B. Cao Q. Peng C. Chen M. Su J. (2024). Clinical efficacy of Zhiyang Xiaozhen granules combined with second-generation antihistamine in the treatment of chronic urticaria. Zhong Nan Da Xue Xue Bao Yi Xue Ban. 49, 175–181. 10.11817/j.issn.1672-7347.2024.230381 38755713 PMC11103067

[B66] Zhao H. (2015). Effect analysis of Yupingfeng combined with Ebastine in the treatment of chronic urticaria. Guangming Zhong Yi 30, 1732–1733.

[B67] Zhou C. (2020). Clinical effect of modified Guizhi Decoction combined with desloratadine citrate in the treatment of chronic idiopathic urticaria. Shi Yong Lin. Chuang Yi Xue 21, 14–15. 10.13764/j.cnki.lcsy.2020.11.005

[B69] Zhuang Y. (2005). Observation on the efficacy of levocetirizine in the treatment of 57 cases of chronic urticaria. Lingnan Pi Fu Xing Bing Ke Za Zhi. 12, 115–116. 10.3969/j.issn.1674-8468.2005.02.015

[B68] Zuberbier T. Aberer W. Asero R. Abdul Latiff A. H. Baker D. Ballmer-Weber B. (2018). The EAACI/GA^2^LEN/EDF/WAO guideline for the definition, classification, diagnosis and management of urticaria. Allergy 73, 1393–1414. 10.1111/all.13397 29336054

