# Supplementary material for: Chinese herbal formulas as adjuncts to antihistamines in chronic spontaneous urticaria: a network meta-analysis of efficacy, recurrence, and safety
Source: Front Pharmacol. 2026 Apr 22;17:1718329. doi: 10.3389/fphar.2026.1718329 (PMC13144053; doi:10.3389/fphar.2026.1718329)
Supplement: Supplementary file 1 [file Supplementaryfile1.zip › Supplementary_materials_for_publication/Supplementary Material and Table/Supplementary Material 1 Search Strategies.docx]

**Supplementary material 1.** Search Strategies

CNKI:

(主题:荨麻疹 + 慢性自发性荨麻疹 +'慢性荨麻疹(cu)'+ 自发性荨麻疹 +特发性荨麻疹 + 慢性荨麻疹)AND(摘要:试验 + 试验硏究 + 临床试验 + 试验分析 +试验结果 +对比试验(精确))

wangfang：

(主题:(荨麻疹) or 主题:(慢性自发性荨麻疹) or 主题:(自发性荨麻疹) or 主题:(特发性荨麻疹) or 主题:(慢性荨麻疹)) and (摘要:(试验) or 摘要:(临床试验) or 摘要:(临床研究) or 摘要:(随机对照研究) or 摘要:(随机对照试验))

VIP：

M=(荨麻疹 OR hives OR urticaria OR 慢性自发性荨麻疹 OR 自发性荨麻疹 OR 特发性荨麻疹 OR 慢性荨麻疹) AND (R =(试验 OR 临床试验 OR 临床研究 OR 随机对照研究 OR 随机对照试验 ) OR M = (试验 OR 临床试验 OR 临床研究 OR 随机对照研究 OR 随机对照试验))

Embase:

('urticaria'/exp OR urticaria OR urticaria:ti,ab,kw) AND 'clinical trial':ti,ab,kw

Pubmed:

((Urticaria[Title/Abstract]) OR (hives[Title/Abstract]) OR (Urticaria[MeSH Terms])) AND ((randomized controlled trial[Text Word]) OR (clinical trial [Text Word]) OR trial[Title/Abstract]))

Chinese Clinical Trial Registry (ChiCTR):

荨麻疹[注册题目]

Cochrane Library:

Urticaria[Title Abstract Keyword]

Clinical Trial:

Urticaria Chronic[Condition/disease]
