# Supplementary material for: Chinese herbal formulas as adjuncts to antihistamines in chronic spontaneous urticaria: a network meta-analysis of efficacy, recurrence, and safety
Source: Front Pharmacol. 2026 Apr 22;17:1718329. doi: 10.3389/fphar.2026.1718329 (PMC13144053; doi:10.3389/fphar.2026.1718329)
Supplement: Supplementary file 1 [file Supplementaryfile1.zip › Supplementary_materials_for_publication/Supplementary Material and Table/Supplementary Material 3 Q statistics to assess homogeneity & consistency.docx]

**FOR SSRI ≥ 90**

Q statistics to assess homogeneity / consistency

Q df p-value

Total 19.85 23 0.6508

Within designs 18.93 22 0.6497

Between designs 0.92 1 0.3364

Design-specific decomposition of within-designs Q statistic

Design Q df p-value

Cetirizine:Loratadine 4.48 3 0.2145

Ebastine:Ebastine+Yupingfeng powder 4.44 3 0.2178

Loratadine:Loratadine+Danggui Yinzi 0.93 1 0.3351

Loratadine:Loratadine+Guizhi decoction 3.09 3 0.3781

Loratadine:Loratadine+Yupingfeng powder 2.23 3 0.5265

Cetirizine:Cetirizine+Yupingfeng powder 3.68 6 0.7193

Cetirizine:Cetirizine+Danggui Yinzi 0.08 3 0.9937

Between-designs Q statistic after detaching of single designs

(influential designs have p-value markedly different from 0.3364)

Detached design Q df p-value

Cetirizine:Loratadine 0.00 0 --

Q statistic to assess consistency under the assumption of

a full design-by-treatment interaction random effects model

Q df p-value tau.within tau2.within

Between designs 0.92 1 0.3364 0 0

**FOR SSRI ≥ 90 (short course)**

Q statistics to assess homogeneity / consistency

Q df p-value

Total 16.15 19 0.6473

Within designs 15.23 18 0.6464

Between designs 0.92 1 0.3364

Design-specific decomposition of within-designs Q statistic

Design Q df p-value

Cetirizine:Loratadine 4.48 3 0.2145

Ebastine:Ebastine+Yupingfeng powder 4.44 3 0.2178

Loratadine:Loratadine+Guizhi decoction 2.95 2 0.2287

Loratadine:Loratadine+Yupingfeng powder 2.23 3 0.5265

Cetirizine:Cetirizine+Yupingfeng powder 1.05 4 0.9023

Cetirizine:Cetirizine+Danggui Yinzi 0.08 3 0.9937

Between-designs Q statistic after detaching of single designs

(influential designs have p-value markedly different from 0.3364)

Detached design Q df p-value

Cetirizine:Loratadine 0.00 0 --

Q statistic to assess consistency under the assumption of

a full design-by-treatment interaction random effects model

Q df p-value tau.within tau2.within

Between designs 0.92 1 0.3364 0 0

**FOR SSRI ≥ 60**

Q statistics to assess homogeneity / consistency

Q df p-value

Total 26.51 17 0.0657

Within designs 25.19 15 0.0475

Between designs 1.32 2 0.5163

Design-specific decomposition of within-designs Q statistic

Design Q df p-value

Cetirizine:Loratadine 8.27 3 0.0408

Ebastine:Ebastine+Yupingfeng powder 7.38 3 0.0608

Loratadine:Loratadine+Yupingfeng powder 1.38 1 0.2410

Loratadine:Loratadine+Guizhi decoction 1.29 1 0.2557

Cetirizine:Cetirizine+Yupingfeng powder 5.94 5 0.3124

Cetirizine:Cetirizine+Danggui Yinzi 0.93 2 0.6268

Between-designs Q statistic after detaching of single designs

(influential designs have p-value markedly different from 0.5163)

Detached design Q df p-value

Cetirizine:Loratadine 0.75 1 0.3875

Cetirizine:Ebastine 0.86 1 0.3532

Cetirizine:Ebastine:Loratadine 0.00 0 --

Q statistic to assess consistency under the assumption of

a full design-by-treatment interaction random effects model

Q df p-value tau.within tau2.within

Between designs 1.09 2 0.5812 0.0783 0.0061

**FOR SSRI ≥ 60 (short course)**

Q statistics to assess homogeneity / consistency

Q df p-value

Total 21.32 14 0.0938

Within designs 20.00 12 0.0672

Between designs 1.32 2 0.5163

Design-specific decomposition of within-designs Q statistic

Design Q df p-value

Cetirizine:Loratadine 8.27 3 0.0408

Ebastine:Ebastine+Yupingfeng powder 7.38 3 0.0608

Loratadine:Loratadine+Yupingfeng powder 1.38 1 0.2410

Cetirizine:Cetirizine+Yupingfeng powder 2.04 3 0.5646

Cetirizine:Cetirizine+Danggui Yinzi 0.93 2 0.6268

Between-designs Q statistic after detaching of single designs

(influential designs have p-value markedly different from 0.5163)

Detached design Q df p-value

Cetirizine:Loratadine 0.75 1 0.3875

Cetirizine:Ebastine 0.86 1 0.3532

Cetirizine:Ebastine:Loratadine 0.00 0 --

Q statistic to assess consistency under the assumption of

a full design-by-treatment interaction random effects model

Q df p-value tau.within tau2.within

Between designs 1.07 2 0.5849 0.0803 0.0064

**FOR AEs**

Q statistics to assess homogeneity / consistency

Q df p-value

Total 15.64 17 0.5495

Within designs 12.95 15 0.6061

Between designs 2.69 2 0.2607

Design-specific decomposition of within-designs Q statistic

Design Q df p-value

Loratadine:Loratadine+Yupingfeng powder 2.56 1 0.1095

Ebastine:Ebastine+Yupingfeng powder 3.36 3 0.3390

Cetirizine:Cetirizine+Yupingfeng powder 4.73 5 0.4492

Loratadine:Loratadine+Guizhi decoction 0.24 1 0.6234

Cetirizine:Loratadine 2.05 5 0.8418

Between-designs Q statistic after detaching of single designs

(influential designs have p-value markedly different from 0.2607)

Detached design Q df p-value

Cetirizine:Ebastine 0.02 1 0.8784

Cetirizine:Loratadine 2.37 1 0.1237

Cetirizine:Ebastine:Loratadine 0.00 0 --

Q statistic to assess consistency under the assumption of

a full design-by-treatment interaction random effects model

Q df p-value tau.within tau2.within

Between designs 2.69 2 0.2607 0 0
