# Supplementary figures and images for: Chinese herbal formulas as adjuncts to antihistamines in chronic spontaneous urticaria: a network meta-analysis of efficacy, recurrence, and safety
Source: Front Pharmacol. 2026 Apr 22;17:1718329. doi: 10.3389/fphar.2026.1718329 (PMC13144053; doi:10.3389/fphar.2026.1718329)

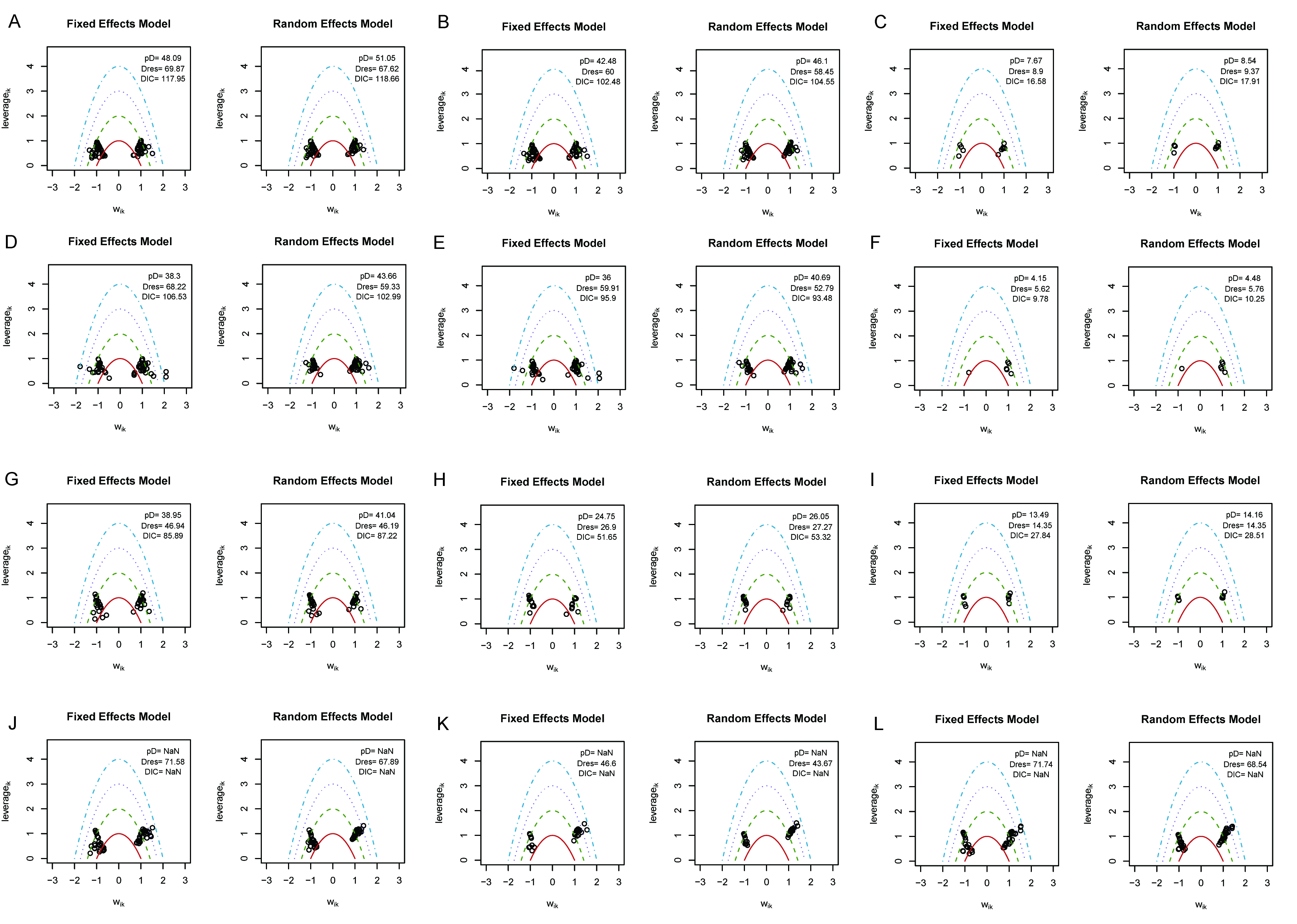

Supplement: Supplementary file 1 [file Supplementaryfile1.zip › Supplementary_materials_for_publication/Supplementary Figure/Fig.S1 Leverage plots and fit statistic accessments for fixed effects model and random effect model.tif]

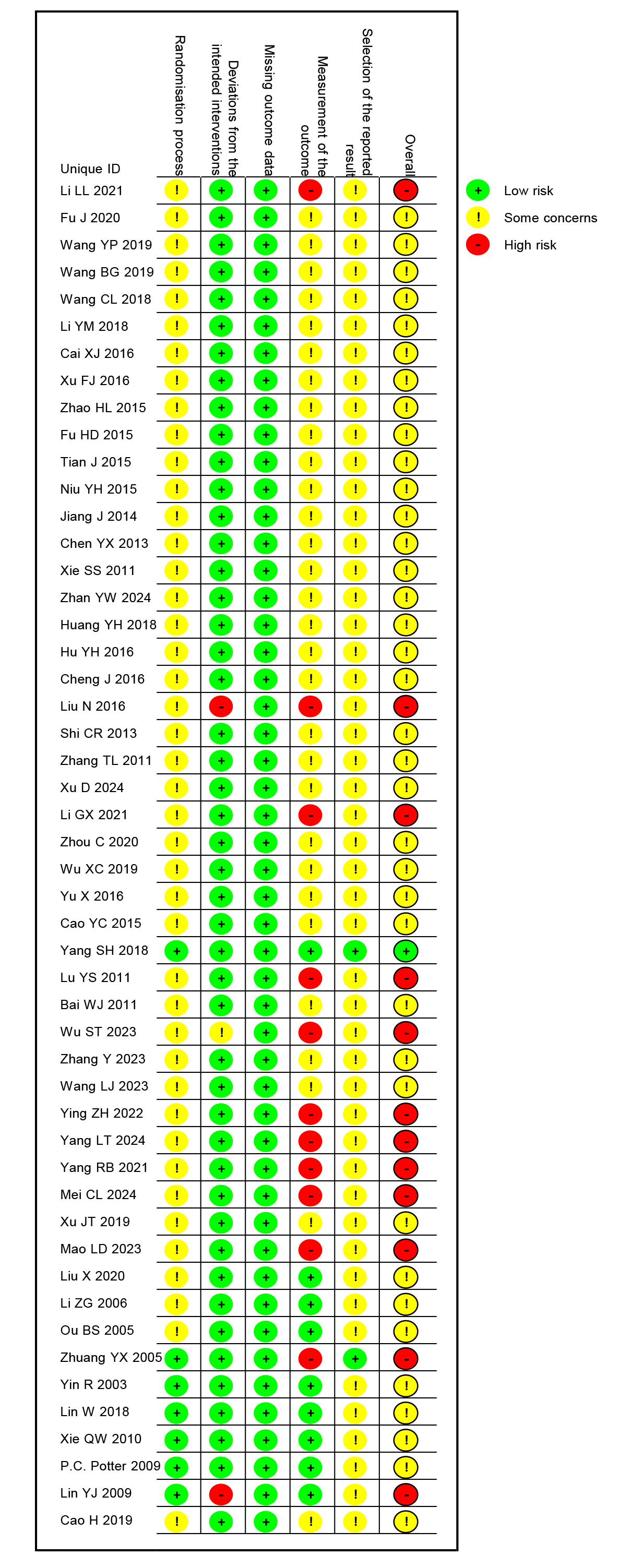

Supplement: Supplementary file 1 [file Supplementaryfile1.zip › Supplementary_materials_for_publication/Supplementary Figure/Fig.S2.Risk of bias for each included study..tif]

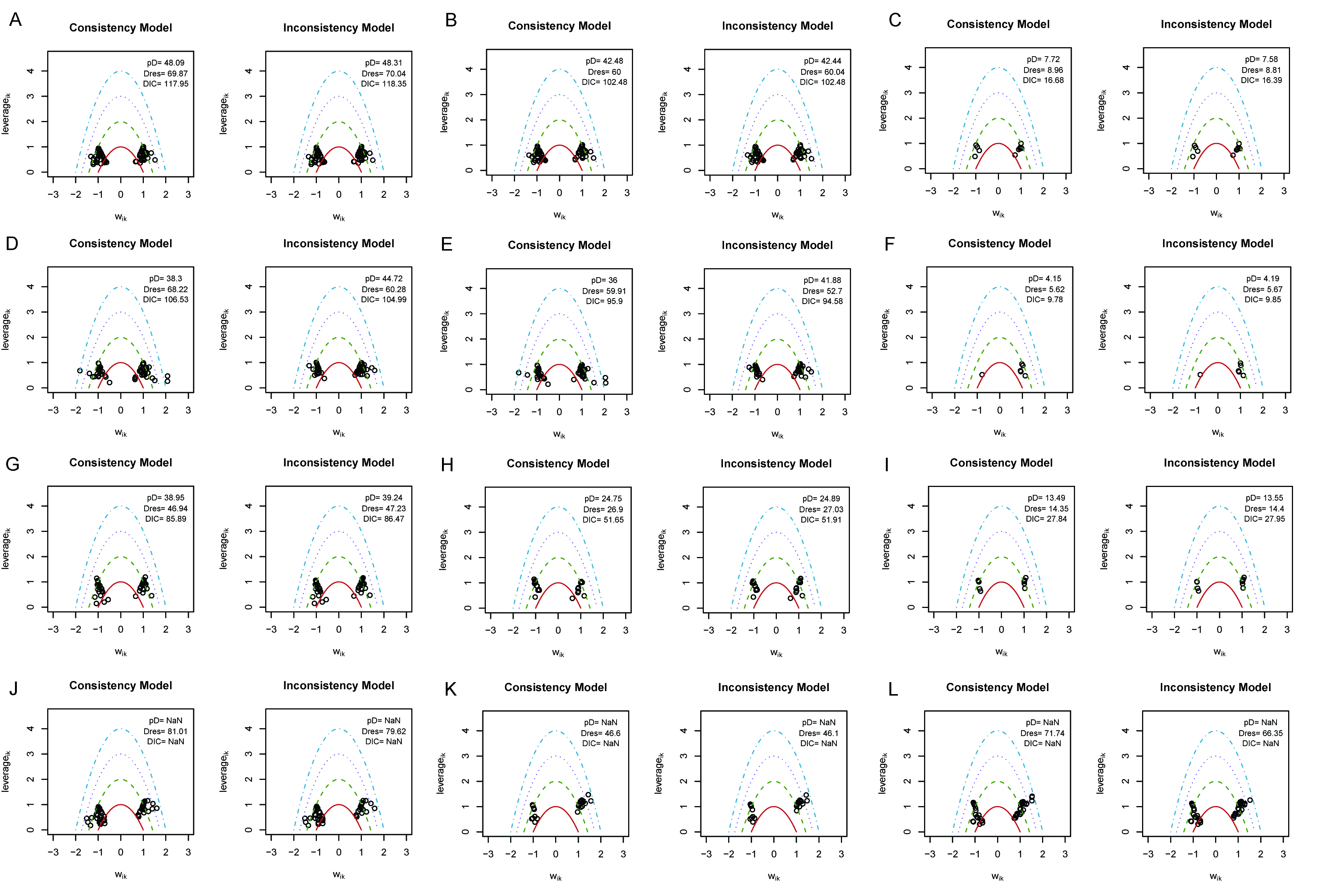

Supplement: Supplementary file 1 [file Supplementaryfile1.zip › Supplementary_materials_for_publication/Supplementary Figure/Fig.S3 Leverage plots and fit statistic accessments for consistency model and inconsistency model.tif]

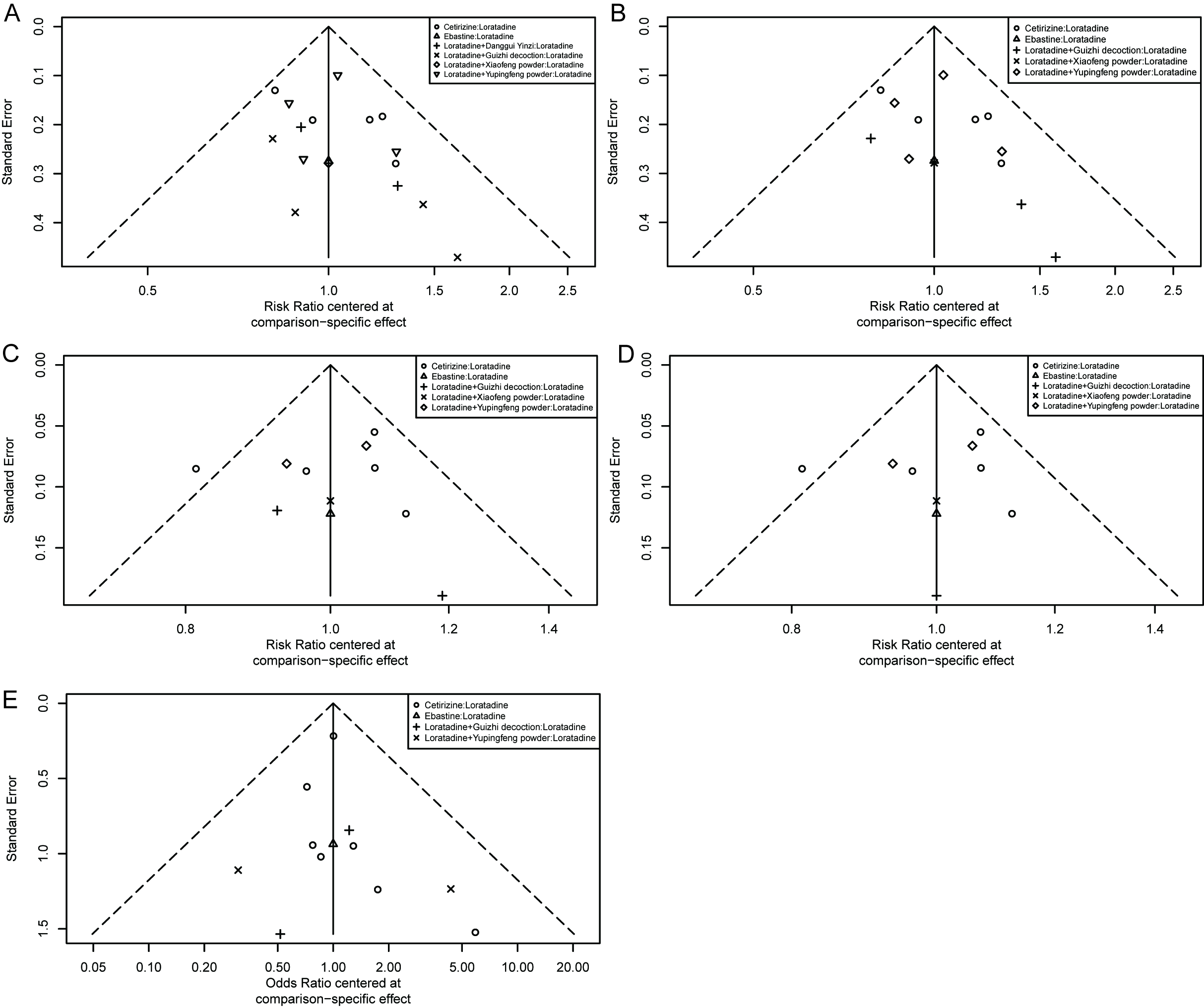

Supplement: Supplementary file 1 [file Supplementaryfile1.zip › Supplementary_materials_for_publication/Supplementary Figure/Fig.S4 Funnel plots.tif]

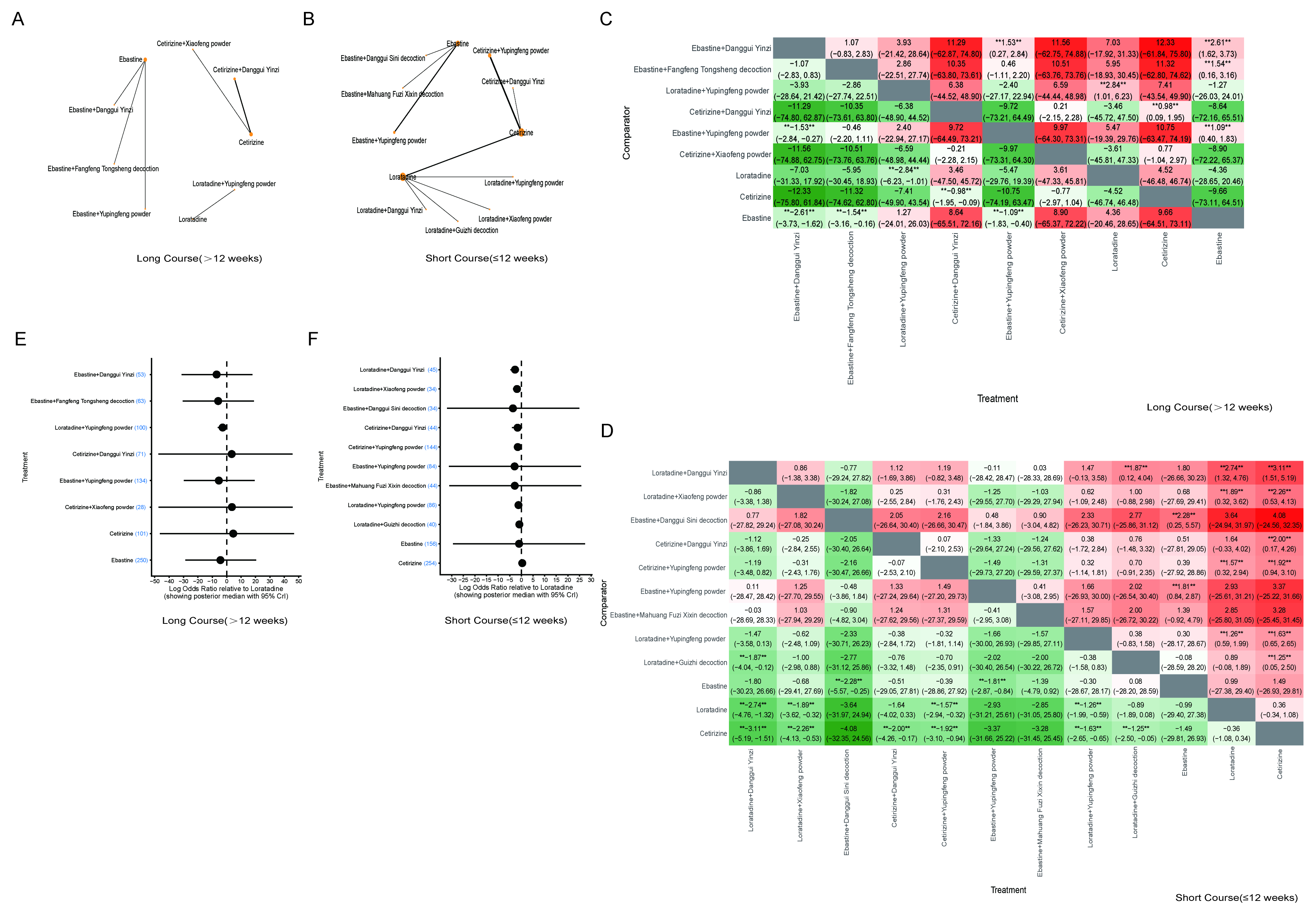

Supplement: Supplementary file 1 [file Supplementaryfile1.zip › Supplementary_materials_for_publication/Supplementary Figure/Fig.S5.Network meta-analysis of recurrence rates in the long-course and short-course treatment subgroups..tif]

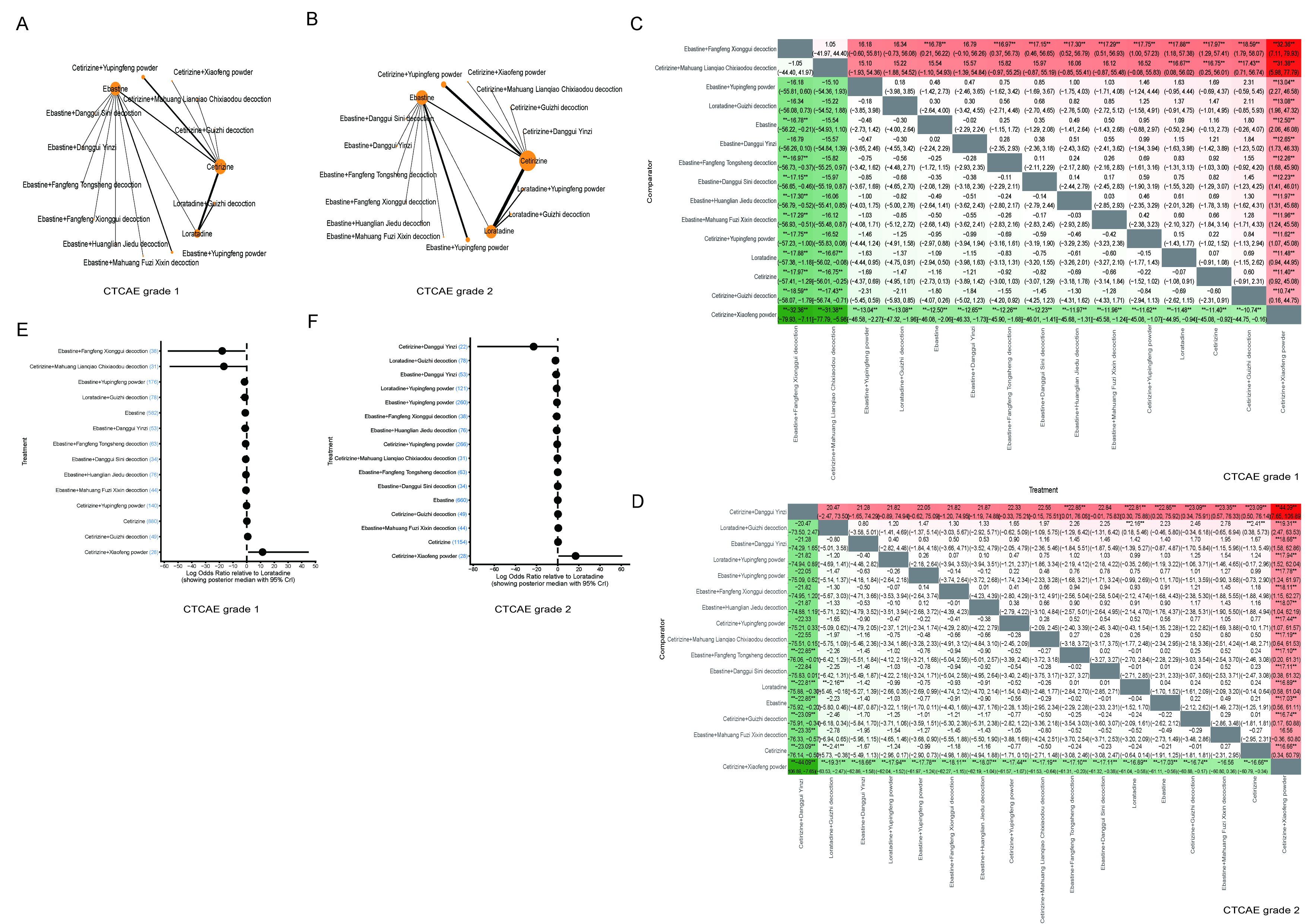

Supplement: Supplementary file 1 [file Supplementaryfile1.zip › Supplementary_materials_for_publication/Supplementary Figure/Fig.S6.Network meta-analysis of adverse events categorized by CTCAE version 5.0 grades.tif]

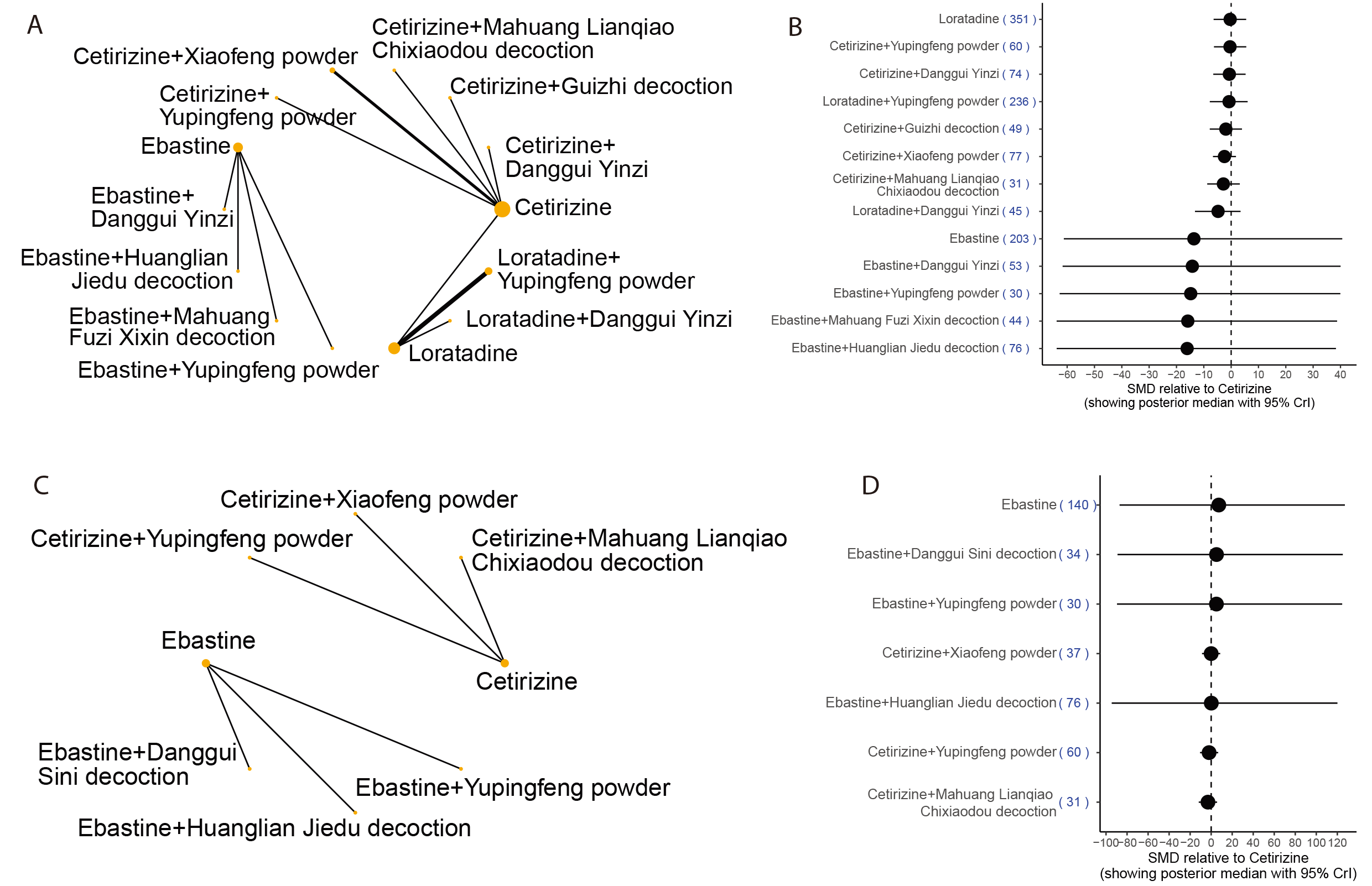

Supplement: Supplementary file 1 [file Supplementaryfile1.zip › Supplementary_materials_for_publication/Supplementary Figure/Fig.S7.Network meta-analysis for laboratory measures..tif]

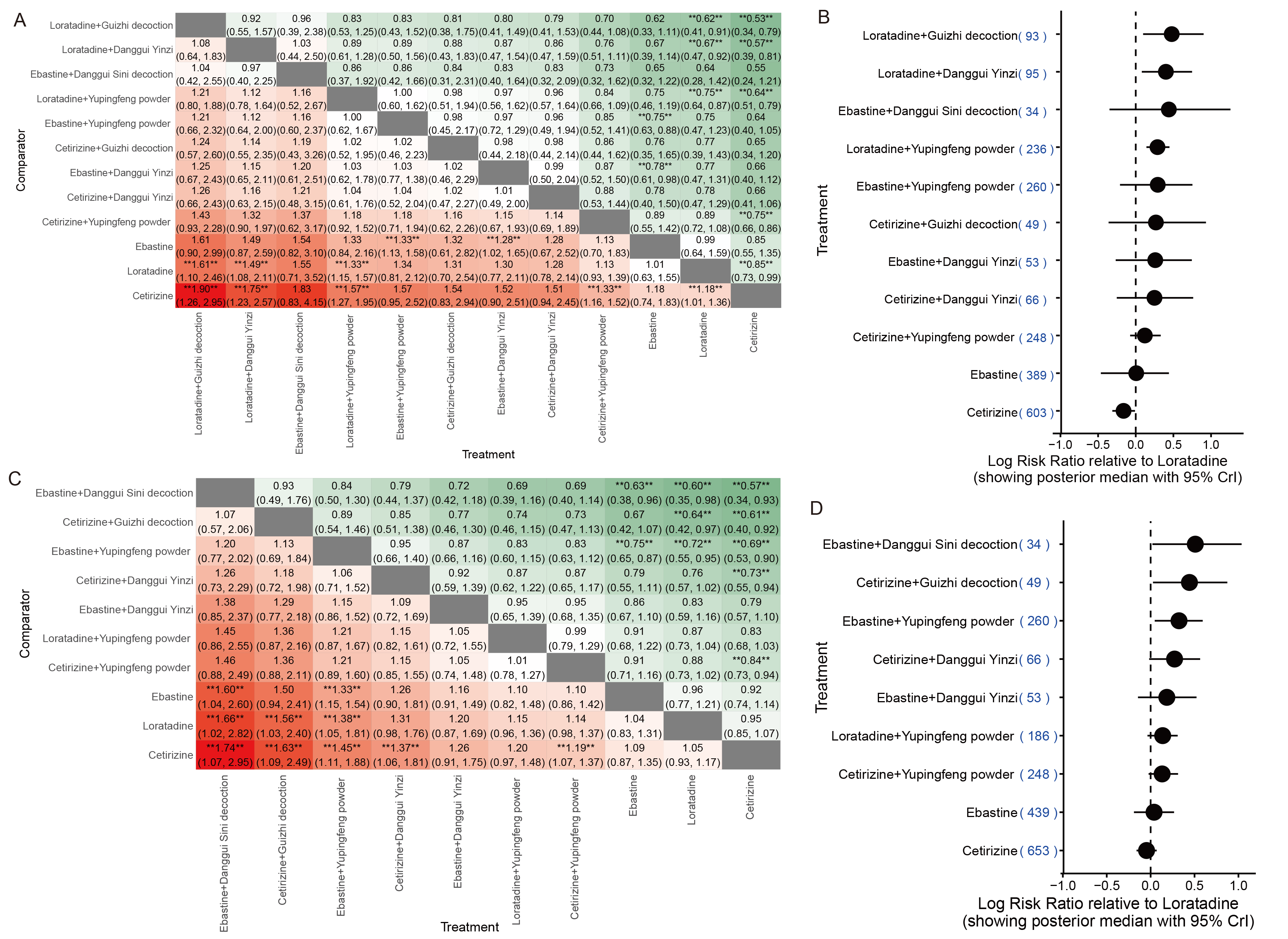

Supplement: Supplementary file 1 [file Supplementaryfile1.zip › Supplementary_materials_for_publication/Supplementary Figure/Fig.S8 Sensitivity analysis accounting for intervention heterogeneity due to syndrome-based modifications of traditional Chinese medicine (TCM).tif]
