# Supplementary material for: Chinese herbal formulas as adjuncts to antihistamines in chronic spontaneous urticaria: a network meta-analysis of efficacy, recurrence, and safety
Source: Front Pharmacol. 2026 Apr 22;17:1718329. doi: 10.3389/fphar.2026.1718329 (PMC13144053; doi:10.3389/fphar.2026.1718329)
Supplement: Supplementary file 1 [file Supplementaryfile1.zip › Supplementary_materials_for_publication/Supplementary Material and Table/Supplementary Appendix Table 5. Summary of adverse events and CTCAE v5.0 severity grading in the included studies.doc]

Supplementary Appendix Table 5. Summary of adverse events and CTCAE v5.0 severity grading in the included studies.

| Study ID | Group | N | Events | Incidence (%) | AEs | CTCAE v5.0 |
| --- | --- | --- | --- | --- | --- | --- |
| LI LL 2021 | Ebastine + *Yupingfeng powder* | 30 |  |  |  | Grade 1 (Mild) |
| LI LL 2021 | Ebastine | 30 |  |  |  | Grade 1 (Mild) |
| Fu J 2020 | Cetirizine + *Yupingfeng powder* | 40 | 1 | 2.5 |  | Grade 1 (Mild) |
| Fu J 2020 | Cetirizine | 40 | 3 | 7.5 |  | Grade 1 (Mild) |
| Wang YP 2019 | Loratadine + *Yupingfeng powder* | 50 |  |  |  | Grade 1 (Mild) |
| Wang YP 2019 | Loratadine | 50 |  |  |  | Grade 1 (Mild) |
| Wang BG 2019 | Loratadine + *Yupingfeng powder* | 35 | 1 | 2.86 | Dizziness | Grade 2 (Moderate) |
| Wang BG 2019 | Loratadine | 35 | 5 | 17.14 | Dizziness, Gastrointestinal discomfort, Somnolence, Fatigue, Dry mouth | Grade 2 (Moderate) |
| Wang CL 2018 | Cetirizine + *Yupingfeng powder* | 60 | 8 | 10 | Gastrointestinal discomfort, Somnolence, Fatigue | Grade 2 (Moderate) |
| Wang CL 2018 | Cetirizine | 60 | 6 | 7 | Gastrointestinal discomfort, Somnolence, Fatigue | Grade 2 (Moderate) |
| Li YM 2018 | Cetirizine + *Yupingfeng powder* | 40 |  |  |  | Grade 1 (Mild) |
| Li YM 2018 | Cetirizine | 40 | 8 |  | Dizziness, Gastrointestinal discomfort, Somnolence, Dry mouth | Grade 2 (Moderate) |
| Cai XJ 2016 | Ebastine + *Yupingfeng powder*s | 60 | 4 |  | Fatigue, Headache | Grade 2 (Moderate) |
| Cai XJ 2016 | Ebastine | 60 | 4 |  | Fatigue, Headache | Grade 2 (Moderate) |
| Xu FJ 2016 | Cetirizine + *Yupingfeng powder* | 40 | 0 | 0 |  | Grade 1 (Mild) |
| Xu FJ 2016 | Cetirizine | 40 | 3 | 7.5 | Dizziness, Gastrointestinal discomfort, Somnolence | Grade 2 (Moderate) |
| Zhao HL 2015 | Ebastine + *Yupingfeng powder* | 134 | 1 |  | Gastrointestinal discomfort | Grade 1 (Mild) |
| Zhao HL 2015 | Ebastine | 134 | 7 |  | Dizziness, Gastrointestinal discomfort | Grade 2 (Moderate) |
| Fu HD 2015 | Loratadine + *Yupingfeng powder* | 100 |  |  |  | Grade 1 (Mild) |
| Fu HD 2015 | Loratadine | 110 |  |  |  | Grade 1 (Mild) |
| Tian J 2015 | Loratadine + *Yupingfeng powder*s | 86 | 2 |  | Dizziness, Somnolence, Delayed response | Grade 2 (Moderate) |
| Tian J 2015 | Loratadine | 86 | 1 |  | Dizziness, Somnolence, Delayed response | Grade 2 (Moderate) |
| Niu YH 2015 | Cetirizine + *Yupingfeng powder* | 64 | 5 | 4.12 | Dizziness, Gastrointestinal discomfort, Somnolence, Fatigue, Dry mouth, Gastrointestinal discomfort | Grade 2 (Moderate) |
| Niu YH 2015 | Cetirizine | 64 | 6 | 7.5 | Dizziness, Gastrointestinal discomfort, Somnolence, Fatigue, Dry mouth, Gastrointestinal discomfort | Grade 2 (Moderate) |
| Jiang J 2014 | Ebastine + *Yupingfeng powder* | 42 | 4 |  | Dizziness, Fatigue, Nausea | Grade 2 (Moderate) |
| Jiang J 2014 | Ebastine | 40 | 3 |  | Dizziness, Somnolence, | Grade 2 (Moderate) |
| Chen YX 2013 | Ebastine + *Yupingfeng powder* | 24 | 4 |  | Dizziness, Gastrointestinal discomfort | Grade 2 (Moderate) |
| Chen YX 2013 | Ebastine | 18 | 6 |  | Dizziness, Somnolence | Grade 2 (Moderate) |
| Xie SS 2011 | Cetirizine + *Yupingfeng powder* | 62 | 1 |  | Somnolence, Dry mouth | Grade 2 (Moderate) |
| Xie SS 2011 | Cetirizine | 60 | 4 |  | Somnolence, Dry mouth, Headache | Grade 2 (Moderate) |
| Zhan YW 2024 | Loratadine + *Danggui Yinzi* | 45 |  |  |  | Grade 1 (Mild) |
| Zhan YW 2024 | Loratadine | 45 |  |  |  | Grade 1 (Mild) |
| Cao H 2019 | Cetirizine + *Danggui Yinzi* | 75 |  |  |  | Grade 1 (Mild) |
| Cao H 2019 | Cetirizine | 75 |  |  |  | Grade 1 (Mild) |
| Huang YH 2018 | Ebastine + *Danggui Yinzi* | 53 | 3 | 5.7 | Somnolence, Dry mouth | Grade 2 (Moderate) |
| Huang YH 2018 | Ebastine | 53 | 5 | 9.4 | Dizziness, Somnolence, Dry mouth | Grade 2 (Moderate) |
| Hu YH 2016 | Cetirizine + *Danggui Yinzi* | 74 |  |  |  | Grade 1 (Mild) |
| Hu YH 2016 | Cetirizine | 70 |  |  |  | Grade 1 (Mild) |
| Cheng J 2016 | Cetirizine + *Danggui Yinzi* | 50 |  |  |  | Grade 1 (Mild) |
| Cheng J 2016 | Cetirizine | 50 |  |  |  | Grade 1 (Mild) |
| Liu N 2016 | Loratadine + *Danggui Yinzi* | 49 |  |  |  | Grade 1 (Mild) |
| Liu N 2016 | Loratadine | 49 |  |  |  | Grade 1 (Mild) |
| Shi CR 2013 | Cetirizine + *Danggui Yinzi* | 25 |  |  |  | Grade 2 (Mild) |
| Shi CR 2013 | Cetirizine | 25 |  |  |  | Grade 3 (Mild) |
| Zhang TL 2011 | Cetirizine + *Danggui Yinzi* | 44 |  |  |  | Grade 2 (Moderate) |
| Zhang TL 2011 | Cetirizine | 44 |  |  |  | Grade 2 (Moderate) |
| Xu D 2024 | Cetirizine + *Guizhi decoction* | 49 | 8 | 16.3 | Dizziness, Gastrointestinal discomfort, Nausea, Vomiting | Grade 2 (Moderate) |
| Xu D 2024 | Cetirizine | 49 | 6 | 12.2 | Dizziness, Gastrointestinal discomfort, Nausea, Vomiting | Grade 2 (Moderate) |
| LI GX 2021 | Ebastine + *Guizhi decoction* | 49 |  |  |  | Grade 2 (Moderate) |
| LI GX 2021 | Ebastine | 49 |  |  |  | Grade 2 (Moderate) |
| Zhou C 2020 | Loratadine + *Guizhi decoction* | 48 | 2 | 4.17 | Dizziness, Dry mouth | Grade 2 (Moderate) |
| Zhou C 2020 | Loratadine | 48 | 6 | 12.5 | Dizziness, Somnolence, Dry mouth | Grade 2 (Moderate) |
| Wu XC 2019 | Loratadine + *Guizhi decoction* | 45 |  |  |  | Grade 1 (Mild) |
| Wu XC 2019 | Loratadine | 44 |  |  |  | Grade 1 (Mild) |
| Yu X 2016 | Loratadine + *Guizhi decoction* | 30 |  |  |  | Grade 1 (Mild) |
| Yu X 2016 | Loratadine | 30 |  |  | Somnolence, Nausea | Grade 2 (Moderate) |
| Cao YC 2015 | Loratadine + *Guizhi decoction* | 40 |  |  |  | Grade 2 (Moderate) |
| Cao YC 2015 | Loratadine | 36 |  |  |  | Grade 2 (Moderate) |
| Yang SH 2018 | Loratadine + *Xiaofeng powder* | 37 | 4 | 10.8 | Sweating, Constipation, Chest tightness, | Grade 2 (Moderate) |
| Yang SH 2018 | Cetirizine + *Xiaofeng powder* placebo | 37 |  |  |  | Grade 1 (Mild) |
| Lu YS 2011 | Loratadine + *Xiaofeng powder* | 40 |  |  |  | Grade 1 (Mild) |
| Lu YS 2011 | Loratadine | 35 |  |  |  | Grade 1 (Mild) |
| Bai WJ 2011 | Loratadine + *Xiaofeng powder* | 34 |  |  |  | Grade 1 (Mild) |
| Bai WJ 2011 | Loratadine | 34 |  |  |  | Grade 1 (Mild) |
| Wu ST 2023 | Cetirizine + *Xiaofeng powder* | 40 |  |  |  | Grade 1 (Mild) |
| Wu ST 2023 | Cetirizine | 40 |  |  |  | Grade 1 (Mild) |
| Zhang Y 2023 | Ebastine + *Maxing Shigan decoction* | 41 |  |  |  | Grade 1 (Mild) |
| Zhang Y 2023 | Ebastine | 41 |  |  |  | Grade 1 (Mild) |
| Wang LJ 2023 | Ebastine + *Danggui Sini decoction* | 34 | 6 | 17.65 | Gastrointestinal discomfort, Dry mouth, Dizziness, High fever，Fatigue | Grade 2 (Moderate) |
| Wang LJ 2023 | Ebastine | 34 | 5 | 14.71 | Gastrointestinal discomfort, Dry mouth, Dizziness, High fever，Fatigue | Grade 2 (Moderate) |
| Ying ZH 2022 | Ebastine + *Fangfeng Tongsheng powder* | 63 | 8 | 12.7 | Gastrointestinal discomfort, Abnormal liver function, Dizziness, Dry mouth | Grade 2 (Moderate) |
| Ying ZH 2022 | Ebastine | 63 | 7 | 11.11 | Gastrointestinal discomfort, Abnormal liver function, Dizziness, Dry mouth | Grade 2 (Moderate) |
| Yang LT 2024 | Ebastine + *Fangfeng Xionggui decoction* | 38 | 1 | 2.63 | Somnolence | Grade 2 (Moderate) |
| Yang LT 2024 | Ebastine | 38 | 3 | 7.89 | Dizziness, Somnolence, Dry mouth | Grade 2 (Moderate) |
| Yang RB 2021 | Ebastine + *Huanglian Jiedu decoction* | 76 | 4 | 5.26 | Gastrointestinal discomfort, Somnolence, Dry mouth | Grade 2 (Moderate) |
| Yang RB 2021 | Ebastine | 76 | 6 | 7.89 | Dizziness, Gastrointestinal discomfort, Somnolence, Dry mouth | Grade 2 (Moderate) |
| Mei CL 2024 | Cetirizine + *Mahuang Lianqiao Chixiaodou decoction* | 31 | 2 | 6.45 | Dizziness, Somnolence | Grade 2 (Moderate) |
| Mei CL 2024 | Cetirizine | 31 | 4 | 12.9 | Dizziness, Gastrointestinal discomfort, Somnolence | Grade 2 (Moderate) |
| Xu JT 2019 | Ebastine + *Mahuang Fuzi Xixin decoction* | 44 | 6 | 13.64 | Dizziness, Somnolence, Dry mouth, Headache, Nausea | Grade 2 (Moderate) |
| Xu JT 2019 | Ebastine | 44 | 4 | 9.09 | Dizziness, Somnolence, Dry mouth, Headache | Grade 2 (Moderate) |
| Mao LD 2023 | Cetirizine + *Chushi Weiling decoction* | 55 |  |  |  | Grade 1 (Mild) |
| Mao LD 2023 | Cetirizine | 55 |  |  |  | Grade 1 (Mild) |
| Liu X 2020 | Ebastine | 50 | 3 | 6 | Fatigue, Dry mouth | Grade 1 (Mild) |
| Liu X 2020 | Cetirizine | 50 | 10 | 20 | Gastrointestinal discomfort, Somnolence., Fatigue, Dry mouth | Grade 2 (Moderate) |
| Li ZG 2006 | Loratadine | 33 | 2 | 6.06 | Dizziness, Somnolence, Dry mouth, Headache | Grade 2 (Moderate) |
| Li ZG 2006 | Cetirizine | 45 | 3 | 6.67 | Dizziness, Somnolence, Dry mouth, Headache | Grade 2 (Moderate) |
| Ou BS 2005 | Cetirizine | 62 | 3 |  | Somnolence, Dry mouth | Grade 2 (Moderate) |
| Ou BS 2005 | Loratadine | 58 |  |  |  | Grade 1 (Mild) |
| Zhuang YX 2005 | Cetirizine | 57 | 7 | 12.28 | Somnolence, Fatigue, Dry mouth | Grade 2 (Moderate) |
| Zhuang YX 2005 | Loratadine | 56 | 8 | 14.29 | Dizziness, Somnolence, Dry mouth | Grade 2 (Moderate) |
| Yin R 2003 | Loratadine | 30 | 2 |  | Dizziness, Somnolence, Dry mouth, Nausea | Grade 2 (Moderate) |
| Yin R 2003 | Cetirizine | 34 | 3 |  | Dizziness, Somnolence, Dry mouth, Nausea | Grade 2 (Moderate) |
| Lin W 2018 | Loratadine | 60 | 1 | 1.67 | Dizziness, Somnolence, Dry mouth | Grade 2 (Moderate) |
| Lin W 2018 | Cetirizine | 60 | 2 | 3.33 | Dizziness, Somnolence | Grade 2 (Moderate) |
| Xie QW 2010 | Loratadine | 50 | 2 |  | Dry mouth, Nausea | Grade 1 (Mild) |
| Xie QW 2010 | Cetirizine | 50 | 2 |  | Dry mouth, Somnolence | Grade 1 (Mild) |
| P.C. Potter 2009 | Cetirizine | 438 | 148 | 33.8 | Fatigue, Dry mouth, Headache, Somnolence | Grade 2 (Moderate) |
| P.C. Potter 2009 | Loratadine | 448 | 131 | 29.2 | Fatigue, Dry mouth, Headache, Somnolence, Diabetes, Myocardial infarction | Grade 3 (Severe) |
| Lin YJ 2009 | Cetirizine | 76 |  |  |  | Grade 2 (Moderate) |
| Lin YJ 2009 | Loratadine | 47 |  |  |  | Grade 2 (Moderate) |
| Note：CTCAE v5.0, Common Terminology Criteria for Adverse Events Version 5.0. | | | | | | |
